# Quantifying phenological landmarks of migration shows nonuniform use of the Caribbean by shorebirds

**DOI:** 10.1002/ece3.9954

**Published:** 2023-04-07

**Authors:** Jessica Rozek Cañizares, Collin B. Edwards, J. Michael Reed

**Affiliations:** ^1^ Department of Biology Tufts University 200 College Ave Medford Massachusetts 02155 USA; ^2^ School of Biological Sciences Washington State University Vancouver Washington USA

**Keywords:** citizen science, full annual cycle, generalized additive models, oversummer, overwinter, phenology

## Abstract

Of the boreal‐ and Arctic‐breeding North American shorebirds that migrate south through the Caribbean, most individuals continue farther south. However, for many species, some individuals remain beyond the southbound migration period (i.e., throughout the temperate winter and/or summer). This variation among individuals adds complexity to observation data, obscures migration patterns, and could prevent the examination of the use of different Caribbean regions by various shorebird species during migration and in the nonmigratory seasons. Here, we present a novel method that leverages a well‐established statistical approach (generalized additive models) to systematically identify migration phenology even for complex passage migrant species with individuals that remain beyond migration. Our method identifies the active migration period using derivatives of a fitted GAM and then calculates phenology metrics based on quantiles of that migration period. We also developed indices to quantify oversummering and overwintering patterns with respect to migration. We analyzed eBird data for 16 North American shorebird species as they traveled South through the insular Caribbean, identifying separate migratory patterns for Cuba, Puerto Rico, Guadeloupe, Aruba, Bonaire, Curaçao, and Trinidad and Tobago. Our results confirm past reports and provide additional detail on shorebird migration in the Caribbean, and identify several previously unpublished regional patterns. Despite Puerto Rico being farther north and closer to continental North America, most species reached Puerto Rico later than other regions, supporting a long‐standing hypothesis that migration strategy (transcontinental vs. transoceanic) leads to geographic differences in migration timing. We also found distinct patterns of migration curves, with some regions and species consistently having either symmetrical or skewed curves; these differences in migration curve shape reflect different migration processes. Our novel method proved reliable and adaptable for most species and serves as a valuable tool for identifying phenological patterns in complex migration data, potentially unlocking previously intractable data.

## Graphical abstract

We developed a method for identifying the active migration period using derivatives of a fitted generalized additive model and then calculated phenology metrics based on quantiles of that migration period. We analyzed eBird data for 16 boreal‐ and arctic‐breeding North American shorebird species as they traveled South through the insular Caribbean. Our results confirm past reports and provide additional detail on shorebird migration in the Caribbean, and identify several previously unpublished regional patterns.

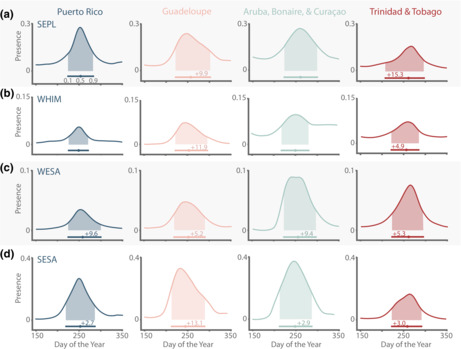

## 1 BACKGROUND

Migratory species are at risk from human‐caused threats, including climate change, habitat loss, and overexploitation (Wilcove & Wikelski, 2008). Because migratory animals depend on a specific set of spatial and temporal resources throughout their journey, there is growing interest in the intersection of the threats they face and their phenology, i.e., the timing of life history events. Accurately describing migration timing is critical for understanding the potential for phenological mismatch, that is, when individuals and resource timing do not coincide (Mayor et al., 2017), mitigating habitat loss (Golet et al., 2018) and informing harvest management policies (Thurber et al., 2020).

Shorebirds are notable for the many species that undertake long‐distance migrations, with some traveling between the extremes of the globe twice annually. During a single calendar year, a migratory bird may rely on a variety of habitats, including arctic, boreal, temperate, and/or tropical regions. Over the last 50 years, shorebird numbers across the globe have declined dramatically (Andres et al., 2012; Brown et al., 2001; Murray et al., 2018). In North America, two‐thirds of shorebird species are in decline (Rosenberg et al., 2019), and climate change is expected to increase their vulnerability (Galbraith et al., 2014). Studies of shorebird phenology have provided insights into stopover ecology (Alexander & Gratto‐Trevor, 1997), migration strategy (Choi et al., 2016), behavioral plasticity (Senner et al., 2019), environmental drivers of phenology (Smith et al., 2020), and the effects of climate change (Galbraith et al., 2014; Saalfeld et al., 2019).

About 78% of North America's temperate‐, boreal‐, and Arctic‐breeding shorebird species spend their nonbreeding season in South American or Caribbean countries (Atlantic Flyway Shorebird Initiative, 2015). The Caribbean provides diverse habitats scattered over 2.75 million km^2^, with some sites known to support significant concentrations of shorebirds (Aguilar et al., 2019; Cañizares & Reed, 2020; Sorenson & Gerbracht, 2014). Shorebirds are both protected and hunted in different parts of the Caribbean, and migration phenology data can be used by resource managers responsible for maintaining habitat for migratory birds (Iglecia & Winn, 2021), and by policymakers that determine hunting season dates.

Despite the importance of understanding the annual cycle of migratory birds (Culp et al., 2017; Hostetler et al., 2015), and the fact that some shorebird species are well‐studied, there is little specific phenological information available regarding their migration through the Caribbean. Many North American shorebirds exhibit an elliptical migration pattern characterized by more eastern southbound movements and more western northbound movements (Cooke, 1910; Gratto‐Trevor & Dickson, 1994; Myers et al., 1987). Thus, in the Caribbean, the highest abundances of migratory shorebirds are observed during the temperate autumn months of southbound migration. The few studies that have investigated seasonal abundances and timing of shorebird migration in this part of the migratory cycle have been conducted mostly in Puerto Rico (e.g., Collazo et al., 1995; Wunderle et al., 1989). Species accounts in Birds of the World (birdsoftheworld.org; accessed 6 May 2021) for several North American shorebirds do not mention the Caribbean at all, despite the species' use of the region.

The quantification of migration phenology has evolved substantially over the last two decades. Classic measures of avian migration phenology include discrete events such as date of first arrival (Cotton, 2003) and date of peak abundance (Chambers et al., 2014). However, such measures are subject to individual variation and population size biases (Edwards & Crone, 2021; Tryjanowski & Sparks, 2001) and do not describe the population‐level phenomenon of migration phenology (Carter et al., 2018; Goodenough et al., 2015; Inouye et al., 2019). One solution is to use quantiles (or percentiles) representing proportions of a population's phenological distribution, as they are unbiased analogs for describing relative timing within the distribution (Bonoan et al., 2021). For example, the 0.05 or 0.1 quantile are phenological landmarks (i.e., identifiable points within the migration distribution) representing initial or early activity, while the 0.9 or 0.95 quantile similarly represents late activity (Jonzén et al., 2006; Newson et al., 2016).

However, current methods are insufficient to characterize the phenology of passage migrants with potentially complex migration curves, particularly when some individuals may be present outside of the migration periods. Our goal is to estimate dates of migration landmarks and identify patterns of southbound migration in a suite of boreal‐ and Arctic‐breeding North American shorebird species (Charadriiformes) as they travel through the insular Caribbean. These shorebird species represent passage migrants and those that spend extended time in the Caribbean (overwintering and/or oversummering). The phenology of shorebirds in the Caribbean is poorly studied compared with their continental ranges and many of these species are of conservation concern.

The most elegant analyses of avian migration to date, have fit logistic models (Hurlbert & Liang, 2012; Mayor et al., 2017) or 4th‐order polynomials (Baillie et al., 2006) to presence data and used inflection points as an estimate of mean arrival date. These approaches may work for temperate regions but are poorly suited to regions where birds are mainly passage migrants, as these models are too restrictive to capture biologically relevant patterns. A useful alternative for modeling the phenology of bird migration is generalized additive models (GAMs) (e.g., Lindén et al., 2017; Newson et al., 2016). GAMs use flexible smoothing functions to generate model estimates that are particularly useful for describing nonlinear patterns (Lindén et al., 2017), though they are underused in the study of bird migration phenology (but see, e.g., Newson et al., 2016).

To conduct our analyses, we used presence data, as available from eBird (Sullivan et al., 2009), as it is a well‐established dataset for modeling migration phenology and it allows in‐depth evaluations of migration timing across broad geographic ranges (e.g., Baillie et al., 2006; Hurlbert & Liang, 2012; Mayor et al., 2017). By using the area under the migration curve of a fitted GAM, we calculated quantile‐based phenological landmarks, analogous to those used in other phenology studies. Quantiles are robust to variation in sample size and model‐fitting, and are suitable for the many ways migration curves can vary (e.g., multimodal, skewed; Edwards & Crone, 2021; Jonzén et al., 2006; Larsen et al., 2022; Newson et al., 2016).

During our investigation, we were motivated to describe shorebird presence beyond southbound migration. Birds that migrate to, and remain in, an area for the winter months are referred to as overwintering. In oversummering, individuals fail to return north to breed and remain on wintering grounds; sometimes this is referred to as deferred migration (Hockey et al., 2018; Martínez‐Curci et al., 2020). In a recent study in Argentina, Navedo and Ruiz (2020) reported high numbers of three Nearctic‐breeding shorebird species oversummering. The authors suggested that conservation strategies for these species may be incomplete as birds can oversummer in sites that are not part of current protection schemes. Thus, having a better understanding of bird presence and habitat use outside of the seasons during which they are most prevalent also may have management applications, particularly for species of conservation concern.

## 2 METHODS

**FIGURE 1:**
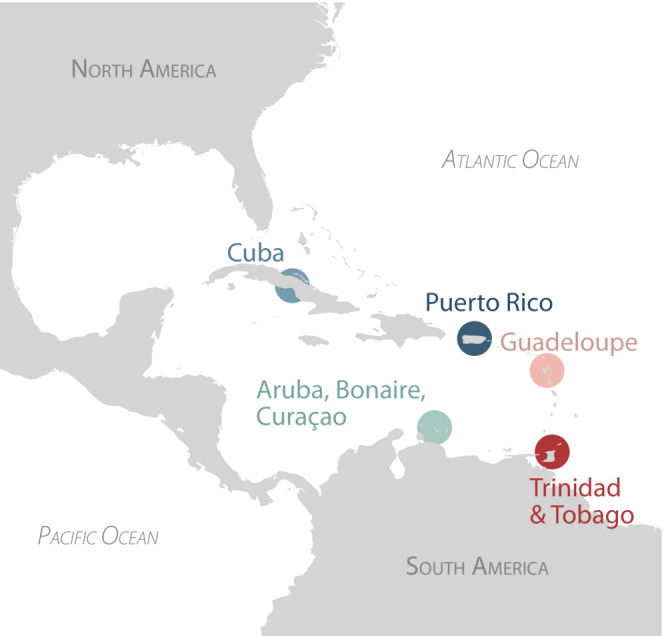
The insular Caribbean consists of thousands of islands and is a key link for birds migrating between North and South America. The five regions included in this study are Cuba, Puerto Rico, Guadeloupe, Aruba, Bonaire, Curaçao, and Trinidad and Tobago.

All data used in this assessment came from the World eBird Basic Dataset (eBird Basic Dataset, 2022; Sullivan et al., 2009). We downloaded all records from eBird on January 13, 2021, and filtered them using the R (R Core Team, 2020) package “Auk” (Strimas‐Mackey et al., 2018) to include only complete, nonduplicate checklists occurring in our regions of interest. We explored five regions in the Caribbean: the islands of Cuba, Puerto Rico, Guadeloupe, and the island groups of Aruba, Bonaire, Curaçao, and Trinidad and Tobago (Figure 1). We restricted our analyses to checklists from January 1, 2010, through December 31, 2020, because eBird reporting has increased substantially through time, and we wanted to focus on a period in time with consistently high reporting. After filtering the eBird Basic Dataset for our study criteria, 1,893,202 bird observations remained for analysis on 138,321 checklists submitted by 6288 unique eBird accounts. Checklist dates were converted to days of the year (DOY) for analyses.

eBird species data are recorded as either presence or raw counts. It is important to acknowledge the possibility of data collection biases that can emerge from using eBird data such as temporal (e.g., counting on weekends or during migration), spatial (e.g., counting inaccessible areas or close to home), or taxonomic biases (e.g., only counting preferred species), and that factors exist that may influence detectability including weather, habitat, and season (Johnston et al., 2014). We assumed that due to a large number of observers, the seasonal, annual, or geographic biases within islands were minimal (Mayor et al., 2017). We also assumed that the proportion of lists reporting shorebirds was consistent over the 11‐year period within islands; we validated this for Puerto Rico (Figure S1). A brief review of Best Practices for Using eBird Data can be found online (Strimas‐Mackey et al., 2020; https://cornelllabofornithology.github.io/ebird‐best‐practices/; accessed 5 January 2021). All filtering and subsequent analyses were conducted with R 4.0.3.

Over 45 species of shorebird have been recorded across the insular Caribbean, though some are rare region‐wide and others frequent only particular islands (Gerbracht & Levesque, 2019). We evaluated 29 migratory shorebird species for inclusion in this analysis within each of the five regions (hereafter “species‐region pair” refers to the unique combination of a species and a region). For all species‐region pairs, we determined the proportion of checklists documenting the presence of each DOY over the 11‐year period. The data were aggregated across years, so the proportion of presence for a species on a given day is the proportion of checklists on that day across the 11 years for which the species of interest was reported. We generally excluded species‐region pairs if a resident breeding population exists in addition to the migratory population, or if there were fewer than 100 presence records across the aggregated year as too few presence records resulted in poor model fits; exceptions were made for WRSA (*Calidris fuscicollis*) in Guadeloupe and Trinidad and Tobago and PESA (*C. melanotos*) in Trinidad and Tobago, each with fewer than 100 presence records but good model fits. The number of checklists per day for each region differed throughout the aggregated year and across regions. For example, in Puerto Rico checklists ranged from 81 to 400 per day, while in Guadeloupe the range was 5 to 145 per day. The regions represent a wide geographical spread across the Caribbean, and each had a mean of at least 40 eBird checklists/day during the DOY window we used to analyze southbound migration (described below). In total, we included 16 species (62 species‐region pairs) in the final analyses. See Table 1 for species and abbreviations used in this manuscript, and Table S1 for species‐region pair inclusion.

**TABLE 1:** Shorebird species included in our analysis and corresponding four‐letter codes.

Common name	Code	Scientific name
Black‐bellied Plover	BBPL	*Pluvialis squatarola*
Semipalmated Plover	SEPL	*Charadrius semipalmatus*
Whimbrel	WHIM	*Numenius phaeopus*
Ruddy Turnstone	RUTU	*Arenaria interpres*
Stilt Sandpiper	STSA	*Calidris himantopus*
Sanderling	SAND	*C. alba*
Least Sandpiper	LESA	*C. minutilla*
White‐rumped Sandpiper	WRSA	*C. fuscicollis*
Pectoral Sandpiper	PESA	*C. melanotos*
Semipalmated Sandpiper	SESA	*C. pusilla*
Western Sandpiper	WESA	*C. mauri*
Short‐billed Dowitcher	SBDO	*Limnodromus griseus*
Spotted Sandpiper	SPSA	*Actitis macularius*
Solitary Sandpiper	SOSA	*Tringa solitaria*
Greater Yellowlegs	GRYE	*T. melanoleuca*
Lesser Yellowlegs	LEYE	*T. flavipes*

### 2.1 Generalized additive model

The proportion of eBird lists that reported presence for each species‐region pair was modeled as a function of DOY using generalized additive models (GAMs) in the mgcv package in R (Wood, 2011). We fitted each species in each region with a separate model, with the proportion of presence as the response variable, and the day of year as a cyclic cubic smooth. We used a binomial error structure, and fit the models using Restricted Maximum Likelihood (REML). Daily proportion of presence data was weighted by a number of checklists per day, making our response equivalent to a binary response with each individual checklist as a separate observation. Following the guidance of Pedersen et al. (2019), model fit was evaluated by ensuring full convergence, visually inspecting residual plots (e.g., Q‐Q plot, histogram of residuals, response vs. fitted values), and examining diagnostic information (e.g., effective degrees of freedom vs. basis dimensions, k‐index) produced using the gam.check() function within mgcv. If serious violations of a model's assumptions were found or the model did not converge, the model for that species region was excluded from further analysis (Kishkinev et al., 2021; McCordic, 2021; Pedersen et al., 2019).

### 2.2 Southbound migration

**FIGURE 2:**
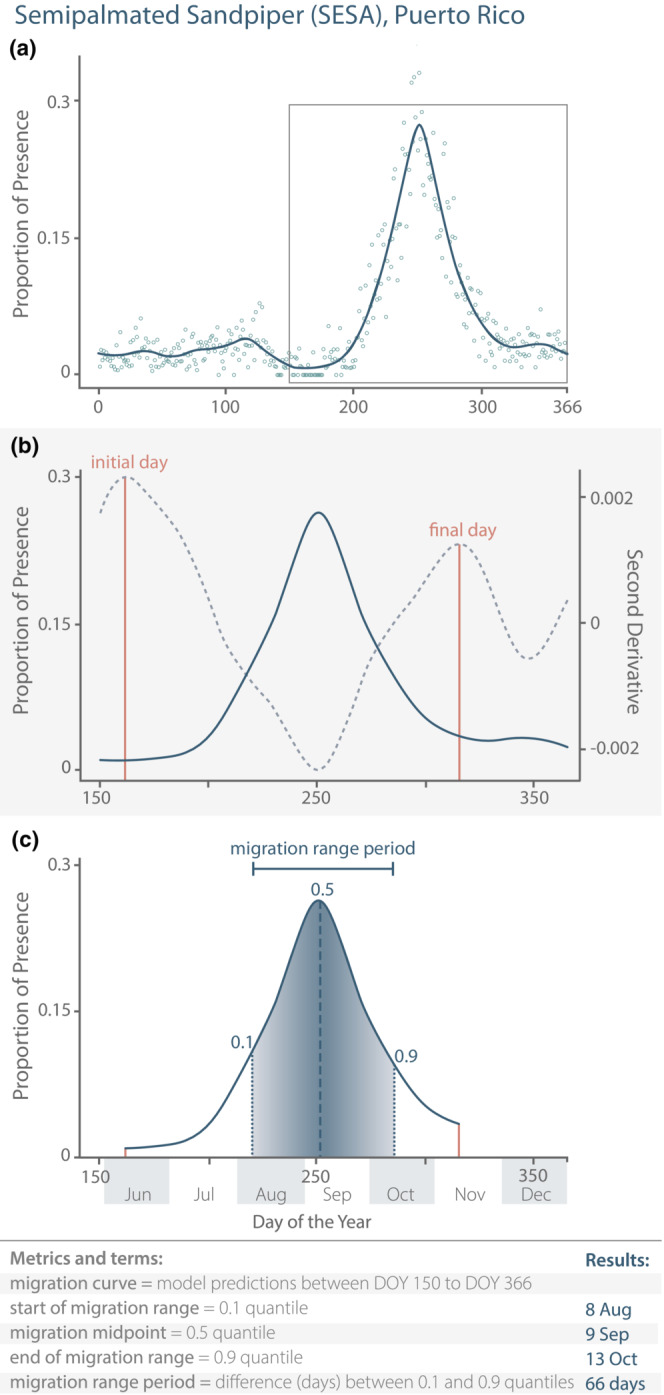
(a) Cumulative (2010–2020) year‐round proportion of presence data (open circles) and fitted GAM (solid line) for the Semipalmated Sandpiper in Puerto Rico, with a box showing DOY 150–366, the range used to evaluate southbound migration. (b) Within the migration window, peaks in the second derivative (dashed line) of the fitted GAM (solid line) were used to estimate the initial and final days (vertical lines) of the southbound migration curve. (c) Using the initial and final days, we calculated the area under the curve between those endpoints to determine the following migration landmarks: start of migration range (0.1 quantile), migration midpoint (0.5 quantile), and end of migration range (0.9 quantile). We calculate the migration range period as the duration between the 0.1 and 0.9 quantiles.

From the fitted models, we examined model predictions of presence for clear southbound (i.e., north‐temperate fall) migration pulses; that is, sudden increases in the proportion of lists reporting each species' presence (Figure S2). The qualitative phenological patterns of migration were obvious in the fitted models for most species, despite the high daily variation and sometimes zero‐presence data even during peak migration that is characteristic of this type of data (Lindén et al., 2017). For species‐region pairs that showed a clear migration pulse, these pulses all fell between DOY 150 (30 May) and the end of the year (Figure 2a). We used the model predictions from DOY 150 to DOY 366 for all further analysis and hereafter refer to the predictions of the model in that period as the “migration curve.”

We wanted an objective, replicable method for determining the start and end points of the migration curve. Intuitively, these correspond to the points in time around migration when the slopes of bird presence changed rapidly (e.g., flat to steep upwards, then steep downwards to flat). Since the second derivative of a function is the change in the slope, peaks in the second derivative represent the beginning of acceleration or ending of deceleration of the original curve (e.g., Figure 2b). We estimated the second derivative of model predictions numerically using finite differences and identified peak values that represented the beginning and end of a migration pulse (Fewster et al., 2000; Newson et al., 2016). Second derivatives are dependent on the shape of the smoothed curve estimated by the GAM and are not guaranteed to capture the true beginning and end of the migration pulse, so we visually inspected each curve to avoid capturing false starts and ends.

Once we established the initial and final days of the southbound migration curve for each species‐region pair, we calculated the area under the curve between those endpoints to determine the following migration landmarks (Figure 2c). The 0.1 quantile (hereafter, the start of migration range) corresponds to the day when 10% of the migration curve has occurred; the 0.5 (hereafter, the migration midpoint) corresponds to the median day of the migration (which differed from the day of the peak if the migration was not symmetric); and the 0.9 quantile (hereafter, the end of migration range) corresponds to the day when 90% of the migration curve has occurred. These specific quantiles are similar to those used in other recent phenology studies (e.g., Bonoan et al., 2021; Edwards & Crone, 2021; Jonzén et al., 2006; Newson et al., 2016). We defined the migration range as the period of time between the start (0.1 quantile) and end (0.9 quantile) of the migration curve (i.e., the period when the middle 80% of presence reports within the migration curve occurred), and the migration range period as the duration (days) between those quantiles (equivalent to the “flight period” reported in some insect phenology studies; Bonoan et al., 2021; Edwards & Crone, 2021). To facilitate others using and building upon our methods, we provide thoroughly commented code for the southbound migration analysis on Figshare (https://doi.org/10.6084/m9.figshare.19406720).

### 2.3 Overwinter and oversummer indices

To describe overwintering and oversummering patterns (i.e., regional use outside of the southbound migration period), we created two indices using the proportion of presence data for the birds included in the previous analysis for the regions of Puerto Rico, Guadeloupe, Aruba, Bonaire, Curaçao, and Trinidad and Tobago. Our indices were inspired by McNeil et al. (1990), which used an oversummering index that corresponded to the number of oversummering individuals as a percentage of the average number of overwintering individuals. As our goal was to create indices for both overwinter and oversummer presence, we compared both to southbound migration. For each species, we determined the mean proportion of eBird lists documenting presence during three 25‐day intervals: DOY 1–25 (winter), DOY 151–175 (summer), and from 12 days before the DOY of the 0.5 quantile identified above to 12 days afterwards (migration). From these mean proportions, we calculated two indices using the mean proportion during migration as a reference: overwintering was the ratio of mean proportions of presence for winter:migration, and oversummering, the summer:migration ratio. An exception to the oversummer index was made for the WRSA in Aruba, Bonaire, and Curaçao, for which summer was calculated for DOY 161–185 instead of DOY 151–175. This was because there was an obvious northbound migration pulse in the temperate spring that resolved by DOY 160 (Figure S3), a pattern that was observed only for this species‐region pair.

The overwinter (winter:migration) and oversummer (summer:migration) indices convey how the mean proportion of presence during winter or during summer compares to the mean proportion of presence during the midpoint of the migratory pulse and serves as a metric for evaluating the relative presence of the region for oversummering and overwintering as compared to migration presence. A value of zero, or close to zero, indicates that a species was rarely recorded in winter or summer relative to its migration midpoint. A value closer to 1 indicates that a species was recorded at similar rates during summer or winter relative to its rate of documentation during migration; that is, a large proportion of the migrants appear to be using the region to overwinter or oversummer. These indices are comparisons within species‐region pairs, and differences between regions could reflect a higher probability to overwinter or oversummer, but also could reflect differences in regional use during migration.

## 3 RESULTS

**FIGURE 3:**
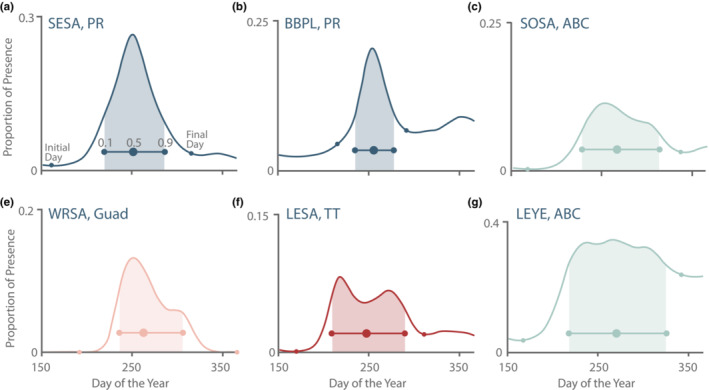
Our methods were able to fit a diversity of migration shapes for different species and locations; here we show a selection of the fitted curve for varying migration patterns. Southbound migration curves (solid line) and migration ranges (i.e., the period of time between the start 0.1 and 0.9 quantiles; shaded area) of six species‐region pairs. Estimated migration landmarks (0.1, 0.5, and 0.9 quantiles) appear on bar within the migration range. Closed circles on migration curve itself represent initial and final day as calculated by the second derivative of the fitted GAM.

The types of curves generated by the models included unimodal (both symmetrical and skewed), multimodal, those that started increasing from, and decreased back, to zero (representing strictly passage migrants), and those that never reached zero, representing species where some individuals oversummer and overwinter (Figure 3). We found that our method of identifying the start and end of the migration curves was flexible enough to be applied successfully to this diversity of curve types. However, there were rare exceptions where the method did not work. For example, even though a migration pulse was evident, in a few cases there were no clear corresponding peaks in the second derivative (e.g., SOSA [*Tringa solitaria*] in Trinidad and Tobago; Figure S2c). There were also two instances of a multimodal migration curve for which we could not determine which peaks should be included: GRYE (*T. melanoleuca*) and SPSA (*Actitis macularius*) in Aruba, Bonaire, and Curaçao; Figure S2d. The species‐region pairs in these cases were not included in the analysis.

### 3.1 Southbound migration

Sixteen shorebird species met our criteria for the analysis of fall migration, that is, birds that had over 100 presence records across the aggregate year, showed a clear migration pulse in the fall, and did not violate model assumptions. Four species met these criteria for Cuba, 16 for Puerto Rico, 16 for Guadeloupe, 13 for Aruba, Bonaire, and Curaçao, and 13 for Trinidad and Tobago.

**FIGURE 4:**
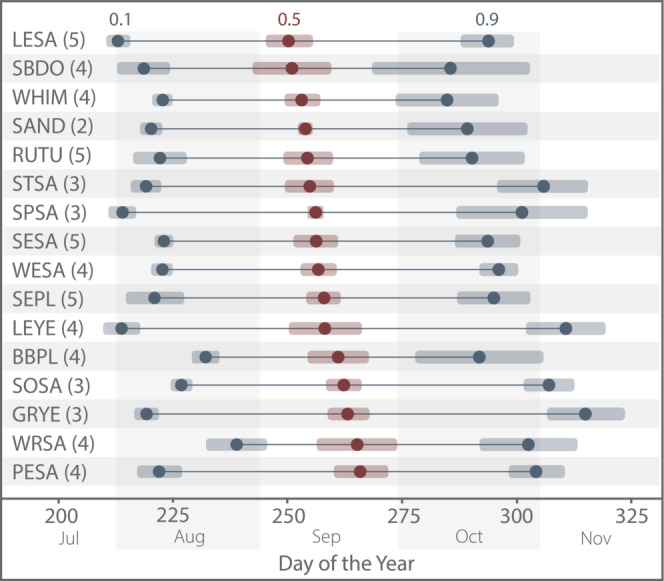
Estimated southbound migration landmarks: start of migration range (0.1 quantile; left blue circle), migration midpoint (0.5 quantile; red circle), and end of migration range (0.9 quantile; right blue circle), averaged across regions by species (shaded area: ± standard deviation). Parenthetical numbers next to species names indicate a number of regions for which southbound migration was analyzed for each species. Species associated with codes are found in Table 1.

Species results for the start and end of the migration range (0.1 and 0.9 quantiles) and the migration midpoint (0.5 quantile), averaged across regions are depicted in Figure 4 (see Table S2 for dates for all 62 species‐region pairs). Migration midpoints occurred almost exclusively in September for all species‐region pairs, with an earlier exception for SBDO in Guadeloupe (30 Aug; all calendar dates are reported as the nonleap year), and a later exception for WRSA in Aruba, Bonaire, and Curaçao (3 Oct). The mean day of the year (DOY) and standard deviation for the migration midpoint for all bird species analyzed per region were as follows: Cuba: 252.2 (9 Sep) ± 2.3 std; Puerto Rico: 254.2 (11 Sep) ± 4.0; Guadeloupe 255.5 (13 Sep) ± 6.8; Trinidad and Tobago 259.8 (17 Sep) ± 6.2; and Aruba, Bonaire, and Curaçao 261.8 (19 Sep) ± 7.2. In general, the start of the migration range for all species occurred in August. Early exceptions were SEPL (*Charadrius semipalmatus*), LESA (*C. minutilla*), SPSA, and LEYE, all in Trinidad and Tobago, which had their start of migration range in fall of July. LESA had the earliest mean start of migration range on 1 Aug (213.0 ± 2.4) followed by LEYE (2 Aug; 213.9 ± 4.1) and SPSA (2 Aug; 214.0 ± 3.0). The species that reached their mean start of migration range at the latest was WRSA at 238.8 (27 Aug) ± 6.4.

**FIGURE 5:**
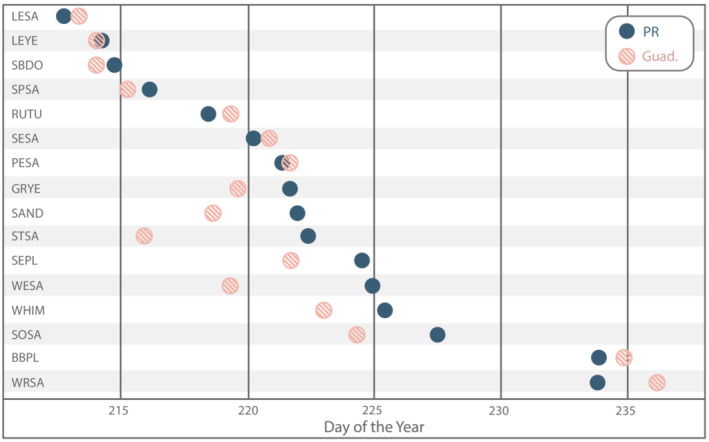
Day of Year (DOY) of the start of migration range (0.1 quantile) of the 16 species that were analyzed for both Puerto Rico (PR, blue circles) and Guadeloupe (pink hashed circles), sorted by date in PR. The start of the migration range occurred in Guadeloupe earlier than in Puerto Rico for nine species, in Puerto Rico earlier for five, and on the same day for two species (LEYE, PESA; DOY differences <0.5 were considered the same day). All data points fall within the month of August (DOY 213–243).

In contrast to what would be expected if birds made their way south through the Caribbean by island‐hopping, we did not see distinct latitudinal patterns of migration landmarks (Figure S4). Instead, we found a surprising pattern when comparing the start of migration range in Puerto Rico, Guadeloupe, Aruba, Bonaire, Curaçao, and Trinidad and Tobago. Despite Puerto Rico being the northmost region and closer to continental North America than the other regions, only three of 16 species (SESA [*C. pusilla*], RUTU [*Arenaria interpres*], and WRSA) had their start of migration range occur in Puerto Rico first (Table S2). Furthermore, the start of the migration range occurred *last* in Puerto Rico for seven species. We were able to directly compare the start of the migration range for all 16 species in Guadeloupe and Puerto Rico as they were the only regions where all 16 species met inclusion criteria. Of these, nine species had their start of migration range occur earlier in Guadeloupe than in Puerto Rico (up to 6.5 days earlier), five occurred in Puerto Rico first, and two occurred on the same day (we treat DOY differences <0.5 as the same day; Figure 5). When including all species‐region pairs in the comparison we found that six species had their start of migration range occur first in Trinidad and Tobago, the southernmost region (Figure S5). As mentioned previously, out of the 62 species‐region pairs, the earliest four, (with the start of the migration range falling in July) occurred in Trinidad and Tobago.

Of the 62 species‐region pairs analyzed, the majority of the end of migration dates occurred in October, though two occurred earlier, in September, and 17 occurred in November. WHIM (*Numenius phaeopus*) had the earliest mean end of migration range at DOY 284.8 (12 Oct) ± 11.2. The latest mean end of migration dates were *Tringa* species: GRYE 315.1 (11 Nov) ± 8.5; LEYE 310.7 (7 Nov) ± 8.7; and SOSA 306.9 (3 Nov) ± 5.5. We again found an unexpected pattern when comparing Puerto Rico to other regions: despite six species having their start of migration range occur last in Puerto Rico, the end of migration range occurred *first* in Puerto Rico for 10 of the 16 species. The species with the longest migration range periods, indicating a more drawn‐out migration, included LEYE (96.3 ± 6.7), GRYE (95.0 ± 9.4), STSA (*C. himantopus*, 86.8 ± 11.3), and SPSA (81.4 ± 14.8 days). Other species had much shorter migration range periods, such as WHIM (61.7 ± 12.0).

**FIGURE 6:**
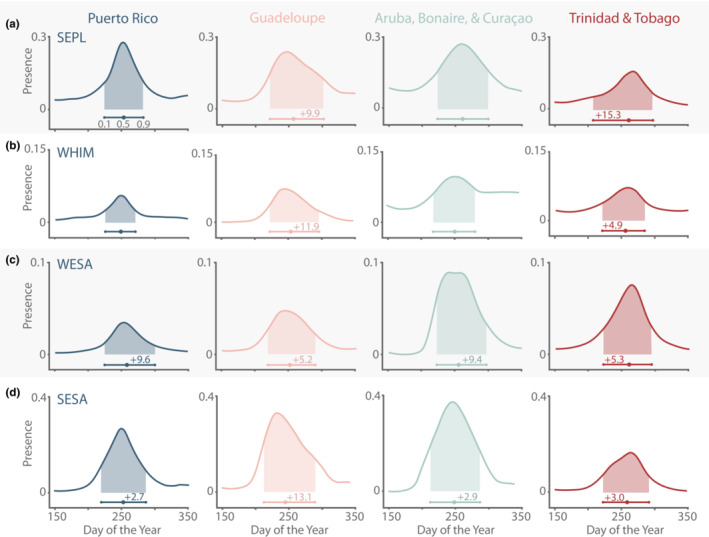
Southbound migration curves and migration landmarks (0.1, 0.5, and 0.9 quantiles) of four species in Puerto Rico, Guadeloupe, Aruba, Bonaire, Curaçao, and Trinidad and Tobago. Shaded areas beneath the model show the migration range. Difference in duration from 0.1 to 0.5 quantiles and from 0.5 to 0.9 displayed on the migration landmark bar as +N and located above the portion with the greater duration (excluded if <2 days). While not the case for all species, many migration curves in Puerto Rico (left column) and Aruba, Bonaire, and Curaçao (third column) tended to be normal or right‐skewed, while those in Guadeloupe (second column) tended to be right‐skewed and those in Trinidad and Tobago (right column) tended to be left‐skewed.

Visual inspection of the shapes of migration curves revealed an unexpected geographic pattern in their shapes. For many species in Guadeloupe, we observed a right‐skewed migration curve with a visibly steeper slope prior to the peak (Figure 6). While less pronounced than in Guadeloupe, we observed right‐skewed migration curves for some species in Aruba, Bonaire, and Curaçao as well. This differed from migration curves of the same species in Puerto Rico models, which generally had a more symmetrical distribution. By contrast, a left‐skewed migration curve was the dominant pattern for southbound migration in Trinidad and Tobago. The right‐skewed migration resulted in a shorter duration between the start of the migration range and the midpoint (0.1–0.5 quantiles) than the midpoint and the end of migration range (0.5–0.9 quantiles), while the left‐skewed distribution resulted in the opposite.

### 3.2 Overwinter and oversummer indices

**FIGURE 7:**
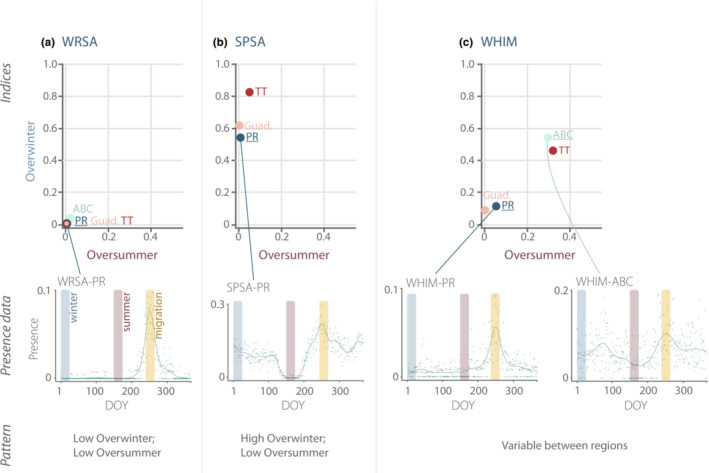
Overwinter and oversummer indices, examples of corresponding presence data, and general patterns by shown for (a) White‐rumped Sandpiper (WRSA); (b) Spotted Sandpiper (SPSA); and (c) Whimbrel (WHIM). The overwinter (winter:migration) and oversummer (summer:migration) indices convey how the mean proportion of presence during winter (DOY 1–25) or during summer (DOY 151–175; WRSA is 161–185) compares to the mean peak proportion of presence during the migration pulse (0.5 quantile DOY ± 9 days). A value close to zero indicates a species rarely recorded in winter or summer, respectively, relative to its migration midpoint. A value closer to 1 indicates a species recorded at similar rates during summer or winter relative to its frequency of being documented during migration. Examples of the corresponding proportion of presence data include presence data (open circles) overlaid with approximate index sampling periods for winter (blue bar), summer (red), and migration (orange). Indices for all regions and species can be found in Figures S6 and S8.

Several patterns emerged across regions. In general, the overwintering indices for species ranged widely, spanning 0 to ~0.8, with 0 representing no individuals recorded in the winter, and 0.8 representing that the proportion of presence in the winter window was approximately 80% of that in the migration period. The oversummering indices, by contrast, typically showed much smaller values (two‐thirds of all indices were <0.1), indicating that the practice was less common. Puerto Rico had the lowest oversummer values (all species below ≤0.16) while Aruba, Bonaire, and Curaçao had the highest, with some species exceeding 0.2 (WHIM, SESA), 0.4 (BBPL; *C. squatarola*) and 0.5 (RUTU; Figure S6). Some species' indices were remarkably consistent across regions. For example, the indices for many *Calidris* sandpipers (WRSA, PESA, SESA, WESA [*C. mauri*]) were always low for both overwinter (0.06 ± 0.08) and oversummer (0.03 ± 0.04; WRSA depicted in Figure 7a), suggesting they primarily used the islands of the Caribbean for migration. Other species, such as SPSA, SOSA, and STSA, had consistently higher overwintering values (SPSA mean 0.67 ± 0.15) but low oversummering values (SPSA mean 0.02 ± 0.03; Figure 7b), suggesting a substantial population remained in the winter, but migrants were generally not present in the summer. By contrast, there were other species whose indices varied between regions. For example, both indices were low for WHIM in Puerto Rico and Guadeloupe, but in Aruba, Bonaire, Curaçao and Trinidad and Tobago, they were five‐ to six‐fold higher (Figure 7c). A similar varying spread was observed for RUTU, BBPL, and LEYE. Complete results for this portion of the analysis by region and by species, with examples linked to the proportion of presence patterns, can be found in the Figures S6–S8.

## 4 DISCUSSION

Accounting for migratory phenology is a key component of species conservation in a changing world. Migration phenology is an important piece of a species' natural history, and phenological shifts can provide information on a species' behavioral plasticity and vulnerability to climate change. The ever‐growing dataset available from eBird provides an opportunity for novel evaluations of bird migration timing across large spatial scales. Several methods have been developed to identify and compare migration landmarks, using eBird or other observational datasets (Baillie et al., 2006; Hurlbert & Liang, 2012; Mayor et al., 2017; Newson et al., 2016; Powers et al., 2021); however, these approaches may not be suitable for passage migrants or those with complex migration curve shapes. We used generalized additive models (GAMs) and their second derivatives to estimate dates of quantile‐based migration. This method is a novel and replicable approach that can be applied both to passage migrants and migrants for which some individuals remain in the region beyond the migratory season. We found that using the second derivative of GAM predictions to identify the start and end of the migration curve was a useful, reliable, and objective way to describe the area under complex curves and extract quantiles. Our case study is the first regional study of migratory shorebird phenology across the Caribbean. The results presented here will be useful for informing local conservation and management planning: helping resource managers plan for foraging and roosting habitat maintenance, providing new information to those undertaking shorebird research activities such as surveys or tagging, and aiding policymakers in harvest season decisions.

Our methods are new and provide a more detailed examination of migratory bird phenology than past studies, but our results are generally consistent with existing published reports. For example, WRSA has been reported with later southbound migration than other Calidrids (Harrington et al., 1991), and they were the species with the latest mean start of migration and migration midpoint in our study. Similarly, LESA is reported as the first shorebirds to peak during southbound migration in the Bay of Fundy, on the east coast of Canada (Hicklin, 1987), and they had the earliest mean start of migration range in our study. However, another study from central Canada found LESA to be later migrants in the fall compared with other species (Alexander & Gratto‐Trevor, 1997). This could indicate different timelines for different flyways, or that this species' relative migration timing varies by region. We found the earliest start of migration range dates occurred at the end of July for four species in Trinidad and Tobago, consistent with reports of July arrivals in nearby Suriname (Spaans, 1978). For the regions where both GRYE and LEYE were included in our analyses (Puerto Rico, Guadeloupe, and Trinidad and Tobago), all migration landmarks occurred earlier for LEYE. This is consistent with reports that peak abundances during fall migration are observed earlier at stopover sites for LEYE compared with GRYE (Tibbitts & Moskoff, 2020). GRYE is also known for its prolonged fall migration period (Elphick & Tibbitts, 2020), and in our study, it had the second‐longest mean migration range period, exceeded only by LEYE. Cooke (1910) noted GRYE's July average arrival times but reported late dates of departure resulting in the nickname “winter yellowlegs.” Wunderle et al. (1989) reported peak abundances of shorebirds on 7 Sep 1985 (DOY 250) at Jobos Bay Estuary in Puerto Rico, and although our study was based on the proportion of presence and not abundance, we found a similar midpoint for all shorebirds analyzed in Puerto Rico of DOY 254.1 (11 Sep) ± 4.0.

When conceptualizing a species migrating south from Arctic‐breeding areas to the Neotropics, it is easy to envision a temporal gradient of movement that corresponds with latitude. However, even within the same species, individuals employ different migration strategies. Some WRSA uses one long‐distance nonstop flight while others travel shorter distances with multiple stopovers (Harrington et al., 1991). Some LEYE follows the coast southward in a series of short “hops,” while others make transoceanic flights (Tibbitts & Moskoff, 2020). In this study, we did not see a latitudinal pattern within species for migration timing landmarks within the (admittedly relatively narrow) latitudinal gradient of the Caribbean. However, we did see an interesting inter‐region pattern with many species' start of migration range occurring later in Puerto Rico, an island that is both farther north and closer to the North American continent, than our other regions. One hypothesis for this pattern is that birds in Guadeloupe, for example, are arriving via a faster transoceanic flight, while those arriving in Puerto Rico made multiple stops on a continental route. Spaans (1978) observed a similar pattern when comparing peak shorebird numbers in Suriname compared with those reported by McNeil (1970) in northeastern Venezuela and proposed the same mechanism.

We observed some general regional patterns in the shape of phenological distributions (Figure 6). Although there was no single shape that represented each region we investigated, the migration curves of several species in Guadeloupe were right‐skewed while those of the same species in Puerto Rico were more symmetrically distributed and in Trinidad and Tobago they were left‐skewed. Some species, such as STSA and WRSA appeared right‐skewed across regions. The shapes of migration curves are driven by a variety of factors including arrival, departure, and turnover rates, and we are unable to definitively identify the factors driving differences between regions (Inouye et al., 2019). However, right‐skewed distributions are consistent with a sudden influx of migrants early in migration, while symmetrical distributions are consistent with a more gradual buildup of migrants. A recent study of SESA suggested that transoceanic migrants are more selective in departure weather than their transcontinental counterparts (Roques et al., 2021). Thus, we speculate that right‐skewed curves represent transoceanic migrants that depart (and then arrive) on a front compared with transcontinental migrants that are less selective about departure conditions. A second possibility is that individuals arrive from different sources (e.g., McDuffie et al., 2022); each origin yields a separate pulse of arrivals but because they overlap temporally, they are not distinct within the overall migration curve and contribute to its skew. Semipalmated Sandpipers that migrate through the Caribbean, for example, originate from eastern Canada to the Alaskan coast (Brown et al., 2017), and we saw the migration curve for this species was right‐skewed in Guadeloupe and left‐skewed in Trinidad and Tobago. A third possible explanation for skewed migration curves is variation in arrival and departure times due to differential migration. Many shorebird species exhibit differential migration, whereby the timing of departure from the breeding grounds differs by sex and age class (Colwell, 2010). This difference in timing could lead to two or more migration peaks (Howe et al., 1989); however, depending on the relative size and timing of the peaks, these peaks might not be distinct and the migration curve may instead appear to be a single skewed peak. These hypotheses are not mutually exclusive and observed patterns may be caused by a combination of these mechanisms.

We believe the shape of migration curves, such as those we observed in our study, could be a valuable tool for investigating shifts due to climate change. There have been calls to better characterize the shape of phenological distributions (Knudsen et al., 2007), and ecologists have recently begun to analyze the shape of migration curves as a tool for investigating underlying biological processes (e.g., Hällfors et al., 2020; Hodgson et al., 2011). Nonuniform changes in the shape of the migration phenology distribution, as opposed to a simple temporal advancement with the same shape, may have population‐ and community‐level consequences not detectable with summary metrics (Carter et al., 2018; Dorian et al., 2020).

Although we only included the dominant migration pulse in our study, we observed that after the obvious September migration pulse there was a second smaller pulse in the proportion of presence in late November/early December for some species‐regions pairs (most notably BBPL and RUTU; Figure S9). Because our data do not include individually marked birds, we can only speculate on the cause(s) of this phenomenon. It is possible that this bimodality is due to differential migration, with the smaller second pulse observed being juveniles and/or late adults that are known to migrate approximately 1 month after the primary wave of adults (Nettleship, 2020; Poole et al., 2020).

One potential application of southbound migration landmarks is to inform local shorebird hunting regulations. For example, in Guadeloupe, open season for shorebird hunting occurs during southbound migration (14 July through the first Sunday in January) and over the past decade the government has implemented several restrictions on shorebird harvest to reduce hunting pressure (Andres, 2017). One tactic has been reducing potential hunting pressure by decreasing the number of days per week harvest is permitted during migration; currently, depending on the date, shorebird hunting is allowed for between 2 and 6 days/week during the open season (Arrêté DEAL/RN n° 971‐2021‐06‐22‐00005). When these regulations are overlaid with the results of our study (Figure S10), we can see that the open season encompasses the entire migration range for all species. However, there are differences in the timing of migration landmarks between species that may result in some species being exposed to greater hunting opportunities (i.e., 6 days/week vs. 2–4). For example, two species had most of their migration range fall within the period of fewer hunting days/week (SBDO, WHIM) while the others, such as BBPL, had about half of its migration range during the most hunting days/week. For BBPL in particular, none of its migration range period occurs during the most restrictive period (2–3 hunting days/week). This study does not address the relationship between hunting days and population dynamics, or if current dates are consistent with the conservation or sustainable use of any species. However, we identified temporal differences in migration landmarks among harvested species; we expect that the timing of hunting set by regulations may benefit some species more than others based on their phenological match (or mismatch) to hunting seasons. Other management applications of shorebird migration phenology data could inform the timing of shorebird surveys, banding activities, or wetland impoundment flooding to allow invertebrate populations to become established (Iglecia & Winn, 2021).

Our study is the first to quantify overwintering and oversummering behaviors for shorebirds across the Caribbean. We used the calculated midpoint (0.5 quantile) of the modeled migration curve to inform an interval from which we extracted the mean proportion of presence and we compared that value to similar means extracted from winter and summer intervals. Results suggest the regular, and sometimes extensive, presence of some shorebird species during winter and summer periods and are consistent with accounts that describe the presence (or absence) of shorebirds in the Caribbean during these times (Billerman et al., 2020; Collazo et al., 1995; Raffaele et al., 1998; Wunderle et al., 1989). Notably, we found that the highest oversummering indices were from species of conservation concern: RUTU and WHIM are of High Concern and BBPL is of Moderate Concern according to the U.S. Shorebird Conservation Plan Partnership (2016). We also found evidence suggesting some species use the Caribbean nonuniformly based on region and season. For example, overwinter and oversummer indices were much higher for WHIM in Aruba, Bonaire, Curaçao, and Trinidad and Tobago than in Puerto Rico or Guadeloupe.

Both overwintering and oversummering are long‐recognized shorebird behaviors and have been reported throughout species' nonbreeding ranges, including the Caribbean (Cooke, 1910). However, oversummering patterns are understudied and the literature tends to focus on determining behavioral drivers (Hockey et al., 2018; Martínez‐Curci et al., 2020; McNeil et al., 1994; Summers et al., 1995) rather than its prevalence (e.g., Navedo & Ruiz, 2020). Oversummering is potentially important because it plays a key role in the development of some juveniles. For example, most first‐year WHIM remains on wintering grounds their first summer, as do some second‐year birds (Skeel & Mallory, 2020), most first‐year SESA oversummer in South America (Hicklin & Gratto‐Trevor, 2020), and it has been posited that many Hudsonian Godwits (*Limosa haemastica*) remain on the nonbreeding grounds until their 3rd or 4th year (Navedo & Ruiz, 2020). Consequently, failure to protect oversummering grounds could impact juvenile cohorts and subsequent population size. Our results can serve as a guide for species and regions that warrant further investigation, such as abundance surveys and seasonal habitat use, as important oversummering habitats may not be included in current conservation schemes (Martínez‐Curci et al., 2020; Navedo & Ruiz, 2020). In particular, our results suggest WHIM in Aruba, Bonaire, Curaçao, and Trinidad and Tobago, and RUTU in Guadeloupe and Aruba, Bonaire, and Curaçao may be worthy of targeted oversummering studies.

We fit GAM to the proportion of checklists for which a species was recorded for each day of the year, rather than bird abundances per se. All else being equal, we expect higher abundance to correspond to increase in the proportion of checklists recording the target species, and past studies have used presence data as a tool to estimate abundance (Conlisk et al., 2009; Gutiérrez et al., 2013). However, there are other factors that could increase the likelihood of encountering a species, such as seasonal changes in the degree of aggregation or habitat selection (Figure S11). Similarly, it is not possible to compare the proportion of checklists reporting a species' presence between regions (i.e., a higher migration peak in one region does not necessarily mean more birds compared to another with a lower value) as the proportion of presence is region‐wide and each region differs in the size, accessibility, and amount of various habitats. The principal findings here are the timing and shapes of the migration curves (which are based on within‐region temporal variation), and the quantiles calculated from these migration curves.

Our models of southbound migration allowed us to estimate when particular migration landmarks occur: start of migration range (0.1 quantile), migration midpoint (0.5 quantile), and end of migration range (0.9 quantile). These quantiles represent the early, middle, and late phases of the southbound migration phenological distribution (Jonzén et al., 2006). We were mindful of the labels we gave the migration landmarks in our study (e.g., “start of migration range”) and to explicitly define them (e.g., “corresponds to the day when 10% of the migration curve had occurred”). We did this because we did not want to conflate the title of the migration landmark with a description of the species' biology. The migration curves in our study are produced from a collection of hundreds or thousands of individual arrivals and departures over time (as described in Inouye et al., 2019). It is not possible to estimate a mean arrival date or end of arrival period from the migration curve, as multiple patterns of arrivals and departures can produce the same migration curve, a point that has been noted in studies of insect phenology (Gross et al., 2007). Although studies employing similar methods often report an estimated categorical or mean arrival date (e.g., Hurlbert & Liang, 2012; Mayor et al., 2017; Powers et al., 2021), these values should actually be interpreted as a descriptive statistic of the fitted model, rather than a description of individual birds. As an illustration, when considering a simple unimodal distribution of the modeled proportion of presence, it is probably not the case that birds continuously arrive over time and once the model peak is reached, no birds arrive and all birds begin to depart. Rather, birds are arriving in varying numbers, staying for varying lengths of time, and departing throughout the duration of the migration curve (Figure S12). Thus, it is entirely possible that arrivals are still occurring in the late phases of migration.

The eBird database serves as a repository for many official bird surveys (e.g., International Shorebird Survey, Caribbean Waterbird Census) and observations from individuals (eBird Basic Dataset, 2022). While the dataset is an important and robust tool for exploring bird presence and phenology across regions (e.g., Hurlbert & Liang, 2012; Mayor et al., 2017), not all observations or local survey efforts are entered into eBird (Cañizares & Reed, 2020). A surprising outcome from this study was how few shorebird species in Cuba met our criteria for the southbound migration analysis. In most cases, this was due to not being able to identify a clear migration pulse. This could be a genuine (lack of) pattern, or it could be due in part to the high seasonal variation in eBird checklists, which are lower during migration (Figure S13)—perhaps due to tourists visiting during the north‐temperate winter. If there are seasonal differences in detectability or types of reporting (e.g., birdwatching tours during some seasons and not others), our model will not be able to disentangle them. The variation in checklist numbers in Cuba was not observed to the same degree in other parts of the Caribbean. An increase in eBird checklists throughout the year, especially during periods of lower checklist numbers (Apr–Nov), may reduce day‐to‐day variability in the proportion of presence data and contribute to successful analyses of this nature in the future. Cuba, however, is an important island for migratory shorebirds and has multiple sites that have recorded significant abundances of a variety of species (Aguilar et al., 2019; Mugica et al., 2006; Nol et al., 2014).

## 5 CONCLUSIONS

The method we describe is a novel combination of existing tools, leveraging the flexibility and robust support for smoothing splines in GAMs with numerically approximating second derivatives to consistently identify the initial and final days of the migration curve, and then using quantiles to identify migration landmarks within that period. This new approach provides a robust and repeatable way to estimate migration landmarks for passage migrants and is flexible enough for migrant species that have individuals present beyond migration. Using our methods, we provide a detailed and thorough description of migratory shorebird phenology across the Caribbean. Our results provide extensive details that supplement existing qualitative information and publications. Our analyses revealed unexpected geographic patterns in the shape of migration curves, the timing of migration landmarks, and presence during nonmigration periods. These patterns indicate that the Caribbean is not used in a uniform way by migratory shorebirds and further research is needed to understand the nuances related to migration timing and geography. The data presented in this paper will be valuable to researchers and to resource managers, conservationists, and policymakers who are responsible for decisions that affect these species, many of which are of conservation concern. We also propose that these data serve as a baseline for future research investigating potential phenological shifts in migratory shorebird southbound migration.

## AUTHOR CONTRIBUTIONS

**Jessica Rozek Cañizares:** Conceptualization (equal); data curation (equal); formal analysis (equal); funding acquisition (equal); investigation (equal); methodology (equal); project administration (equal); software (equal); visualization (lead); writing – original draft (equal); writing – review and editing (equal). **Collin B. Edwards:** Conceptualization (equal); data curation (equal); formal analysis (equal); investigation (equal); methodology (equal); project administration (equal); resources (equal); software (equal); supervision (supporting); visualization (supporting); writing – original draft (equal); writing – review and editing (equal). **J. Michael Reed:** Conceptualization (equal); data curation (supporting); formal analysis (supporting); funding acquisition (equal); investigation (equal); methodology (equal); project administration (equal); resources (equal); software (equal); supervision (lead); visualization (supporting); writing – original draft (equal); writing – review and editing (equal).

## FUNDING INFORMATION

This work was funded in part by the Tufts University Department of Biology and the PEO Scholar Award to JRC. This work was partly completed while CBE was funded by SERDP RC2700. Funding for publication was made available from the Tufts University Tisch Library Open Access Publishing Fund.

## CONFLICT OF INTEREST STATEMENT

The authors declare that they have no competing interests.

## Supporting information


Appendix S1

## Data Availability

The datasets generated and/or analyzed during the current study are available in the Figshare repository (https://doi.org/10.6084/m9.figshare.19406720).
