# Supplementary material for: Quantifying phenological landmarks of migration shows nonuniform use of the Caribbean by shorebirds
Source: Ecol Evol. 2023 Apr 7;13(4):e9954. doi: 10.1002/ece3.9954 (PMC10082156; doi:10.1002/ece3.9954)
Supplement: Supplementary file 1 — Appendix S1 [file ECE3-13-e9954-s001.pdf]

Appendix

This appendix accompanies the article "Quantifying phenological landmarks of migration shows non-uniform use of the Caribbean by shorebirds " by Cañizares, Edwards, and Reed in *Ecology and Evolution*.

Cuba
PR - Puerto Rico
Guad. - Guadeloupe
ABC - Aruba, Bonaire, & Curaçao
TT - Trinidad & Tobago

Figure S1 Checklists reporting shorebirds in Puerto Rico........2

Table S1 Species-region pair inclusion........3

Figure S2 Examples of presence and absence of a clear migration pulse........4

Figure S3 White-rumped Sandpiper year-round data........5

Table S2 Southbound migration landmarks........6

Figure S4 Migration landmarks by species and approximate lattitude........7

Figure S5 Start of Migation Range (0.1 Quantile) Across Regions........8

Figure S6 Overwinter and oversummer indices by region........9

Figure S7 Examples of corresponding year-round presence data with overwinter and oversummer indices........10

Figure S8 Overwinter and oversummer indices by species........11

Figure S9 Examples of presence and absence of end-of-year secondary model peaks........12

Figure S10 Southbound migration and hunting season in Guadeloupe........13

Figure S11 Examples of potential underlying phenomena affecting proportion of presence........14

Figure S12 Individual and group contributions to migration curve........15

Figure S13 Total eBird Checklists per DOY (2010-2020) by region........16

Figure S1 Checklists reporting shorebirds in Puerto Rico

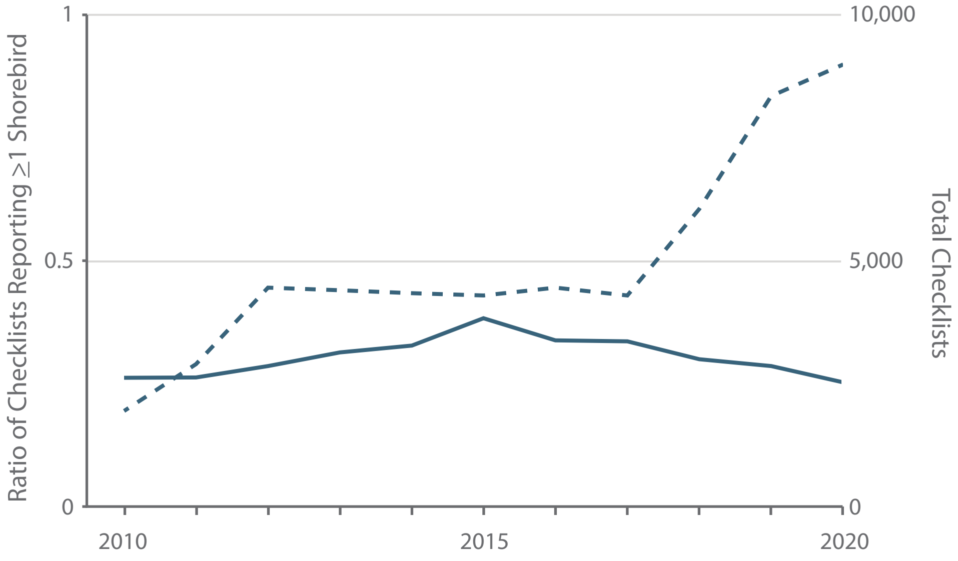

Figure S1 From 2010-2020, the ratio of eBird checklists reporting at least one shorebird stayed consistent (0.31 ± 0.04; solid line) in Puerto Rico despite the 4.5-fold increase in total checklists over the 11-year period (dashed line).

Table S1 Species-region pair inclusion

	Cuba	PR	Guad.	ABC	TT
Black-necked Stilt (BNST)<br>*Himantopus mexicanus*					
American Avocet (AMAV)<br>*Recurvirostra americana*					
American Oystercatcher (AMOY)<br>*Haematopus palliatus*					
Black-bellied Plover (BBPL)<br>*Pluvialis squatarola*		SM	SM	SM	SM
American Golden-Plover (AMGP)<br>*P. dominica*					
Snowy Plover (SNPL)<br>*Charadrius nivosus*					
Wilson's Plover (WIPL)<br>*C. wilsonia*					
Semiplamated Plover (SEPL)<br>*C. semipalmatus*	SM	SM	SM	SM	SM
Piping Plover (PIPL)<br>*C. melodus*					
Killdeer (KILL)<br>*C. vociferus*					
Upland Sandpiper (UPSA)<br>*Bartramia longicauda*					
Whimbrel (WHIM)<br>*Numenius phaeopus*		SM	SM	SM	SM
Hudsonian Godwit (HUGO)<br>*Limosa haemastica*					
Ruddy Turnstone (RUTU)<br>*Arenaria interpres*	SM	SM	SM	SM	SM
Red Knot (REKN)<br>*Calidris canutus*					
Stilt Sandpiper (STSA)<br>*C. himantopus*		SM	SM	SM	
Sanderling (SAND)<br>*C. alba*		SM	SM		
Least Sandpiper (LESA)<br>*C. minutilla*	SM	SM	SM	SM	SM
White-rumped Sandpiper (WRSA)<br>*C. fuscicollis*		SM	SM	SM	SM
Pectoral Sandpiper (PESA)<br>*C. melanotos*		SM	SM	SM	SM
Semipalmated Sandpiper (SESA)<br>*C. pusilla*	SM	SM	SM	SM	SM
Western Sandpiper (WESA)<br>*C. mauri*		SM	SM	SM	SM
Short-billed Dowitcher (SBDO)<br>*Limnodromus griseus*		SM	SM	SM	SM
Wilson's Phalarope (WIPH)<br>*Phalaropus tricolor*					
Spotted Sandpiper (SPSA)<br>*Actitis macularius*		SM	SM		SM
Solitary Sandpiper (SOSA)<br>*Tringa solitaria*		SM	SM	SM	
Greater Yellowlegs (GRYE)<br>*T. melanoleuca*		SM	SM		SM
Willet (WILL)<br>*T. semipalmata*					
Lesser Yellowlegs (LEYE)<br>*T. flavipes*		SM	SM	SM	SM

Table S1 Results of inclusion criteria for 29 shorebird species records in 5 regions. SM = species included for southbound migration analysis.

Figure S2 Examples of presence and absence of a clear migration pulse

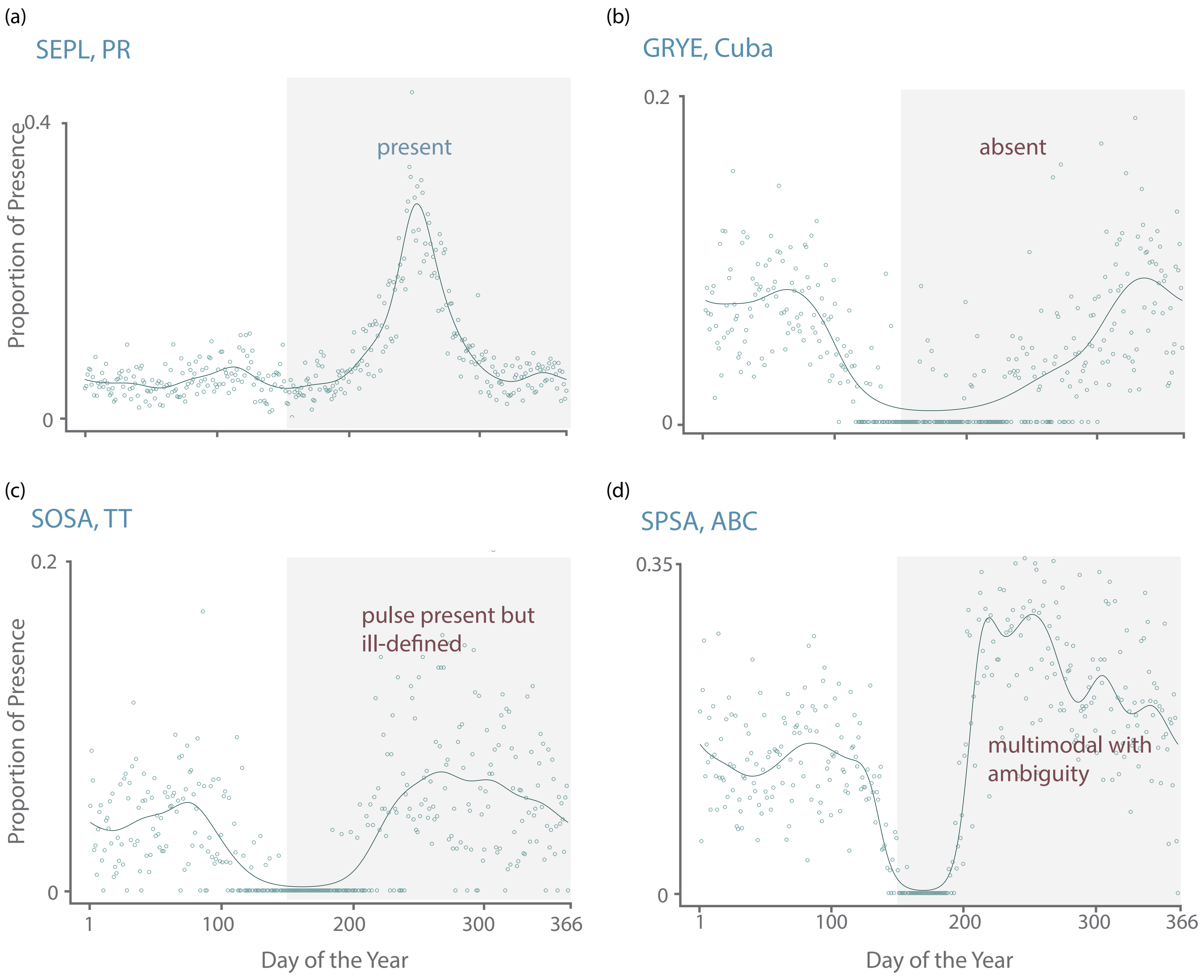

Figure S2 Cumulative (2010-2020) year-round proportion of presence data (open circles) and fitted GAM (solid line). Gray box indicates DOY 150-366, the period for which model predictions were used for southbound migration analysis. A clear migration pulse (as depicted in (a)) was necessary to perform the analysis. A migration pulse could be (b) absent, (c) present but ill-defined if we were not able to determine initial and final days by the second derivative, or in two cases, (d) present but the multimodal nature of the migration curve prevented unambiguous identification of the initial and final days. Thus, such a models (b-d) would not be included in our analysis.

Figure S3 White-rumped Sandpiper year-round data

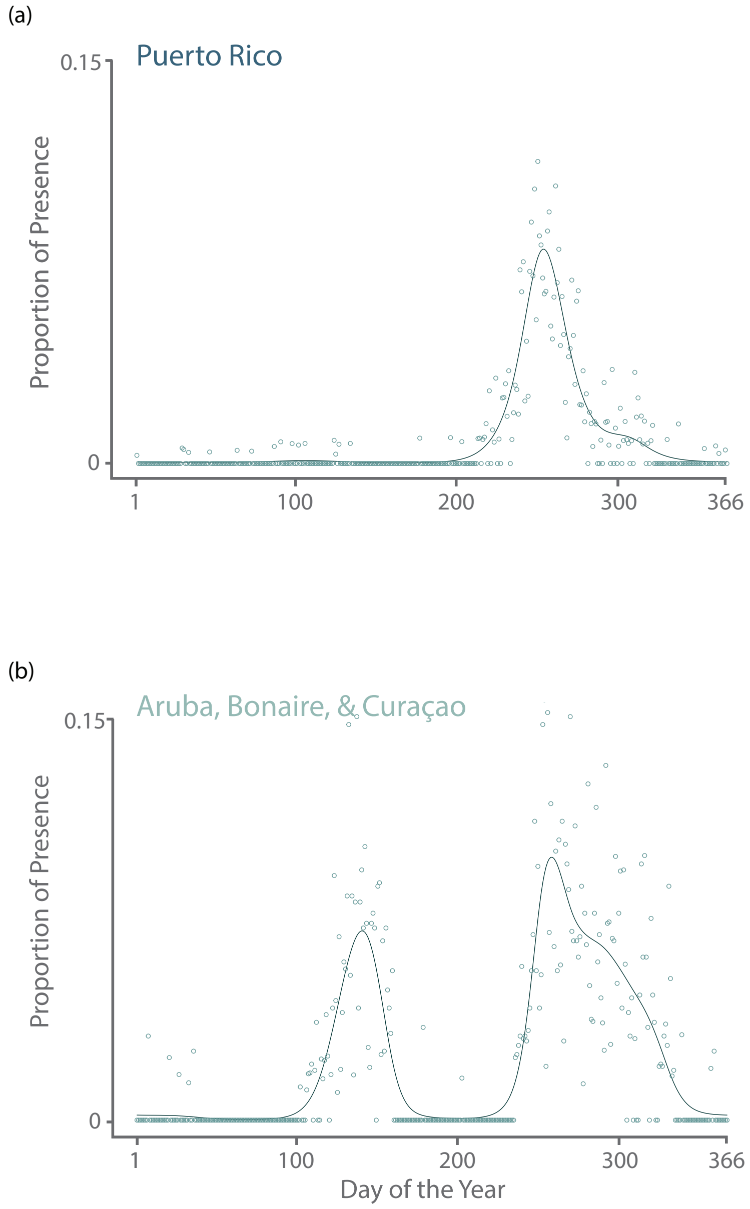

Figure S3 Cumulative (2010-2020) year-round proportion of presence data (open circles) and fitted GAM (solid line) for WRSA in PR (a) and ABC (b). A clear southbound migration pulse appears in both regions while a northbound migration pulse appears only in ABC.

Table S2 Southbound migration landmarks

	Cuba			Puerto Rico			Guadeloupe			Aruba, Bonaire, & Curaçao			Trinidad & Tobago			Migration Range Period ± std (days)
	0.1	0.5	0.9	0.1	0.5	0.9	0.1	0.5	0.9	0.1	0.5	0.9	0.1	0.5	0.9	
Black-bellied Plover (BBPL) *Pluvialis squatarola*	—	—	—	22 Aug	12 Sep	4 Oct	23 Aug	25 Sep	5 Nov	16 Aug	13 Sep	12 Oct	20 Aug	22 Sep	24 Oct	59.7 ± 13.1
Semiplamated Plover (SEPL) *Charadrius semipalmatus*	13 Aug	12 Sep	18 Oct	13 Aug	11 Sep	10 Oct	10 Aug	14 Sep	29 Oct	12 Aug	18 Sep	26 Oct	29 Jul	19 Sep	27 Oct	74.0 ± 12.3
Whimbrel (WHIM) *Numenius phaeopus*	—	—	—	13 Aug	5 Sep	28 Sep	11 Aug	11 Sep	25 Oct	8 Aug	10 Sep	11 Oct	11 Aug	15 Sep	15 Oct	61.9 ± 12.3
Ruddy Turnstone (RUTU) *Arenaria interpres*	9 Aug	7 Sep	17 Oct	7 Aug	7 Sep	2 Oct	7 Aug	13 Sep	3 Nov	8 Aug	11 Sep	16 Oct	21 Aug	20 Sep	18 Oct	68.1 ± 12.5
Stilt Sandpiper (STSA) *Calidris himantopus*	—	—	—	10 Aug	13 Sep	25 Oct	4 Aug	6 Sep	29 Oct	7 Aug	16 Sep	13 Nov	—	—	—	86.6 ± 11.2
Sanderling (SAND) *C. alba*	—	—	—	10 Aug	10 Sep	7 Oct	7 Aug	12 Sep	25 Oct	—	—	—	—	—	—	69.0 ± 15.3
Least Sandpiper (LESA) *C. minutilla*	1 Aug	9 Sep	19 Oct	1 Aug	6 Sep	16 Oct	1 Aug	2 Sep	21 Oct	4 Aug	15 Sep	30 Oct	29 Jul	5 Sep	17 Oct	80.7 ± 3.7
White-rumped Sandpiper *C. fuscicollis* (WRSA)	—	—	—	22 Aug	12 Sep	17 Oct	24 Aug	19 Sep	1 Nov	5 Sep	3 Oct	11 Nov	25 Aug	24 Sep	27 Oct	63.8 ± 5.9
Pectoral Sandpiper (PESA) *C. melanotos*	—	—	—	9 Aug	14 Sep	24 Oct	10 Aug	20 Sep	28 Oct	16 Aug	28 Sep	7 Nov	4 Aug	21 Sep	28 Oct	82.3 ± 4.0
Semipalmated Sandpiper *C. pusilla* (SESA)	11 Aug	8 Sep	11 Oct	8 Aug	9 Sep	13 Oct	9 Aug	10 Sep	25 Oct	13 Aug	18 Sep	27 Oct	11 Aug	17 Sep	20 Oct	70.6 ± 6.5
Western Sandpiper (WESA) *C. mauri*	—	—	—	13 Aug	15 Sep	28 Oct	7 Aug	9 Sep	18 Oct	11 Aug	13 Sep	25 Oct	11 Aug	19 Sep	23 Oct	73.5 ± 2.1
Short-billed Dowitcher *Limnodromus griseus* (SBDO)	—	—	—	3 Aug	4 Sep	30 Sep	2 Aug	30 Aug	5 Oct	14 Aug	19 Sep	7 Nov	7 Aug	9 Sep	8 Oct	67.0 ± 11.7
Spotted Sandpiper (SPSA) *Actitis macularius*	—	—	—	4 Aug	15 Sep	2 Nov	3 Aug	13 Sep	8 Nov	—	—	—	30 Jul	11 Sep	12 Oct	87.1 ± 11.6
Solitary Sandpiper (SOSA) *Tringa solitaria*	—	—	—	16 Aug	17 Sep	31 Oct	12 Aug	17 Sep	31 Oct	17 Aug	24 Sep	9 Nov	—	—	—	80.1 ± 4.2
Greater Yellowlegs (GRYE) *T. melanoleuca*	—	—	—	10 Aug	17 Sep	4 Nov	8 Aug	19 Sep	9 Nov	—	—	—	4 Aug	25 Sep	21 Nov	95.8 ± 11.2
Lesser Yellowlegs (LEYE) *T. flavipes*	—	—	—	2 Aug	13 Sep	2 Nov	2 Aug	11 Sep	3 Nov	7 Aug	27 Sep	20 Nov	28 Jul	11 Sep	2 Nov	96.9 ± 6.1

Table S2 Corresponding dates of estimated start of migration range (0.1 quantile), migration midpoint (0.5 quantile), and end of migration range (0.9 quantile) rounded to the nearest DOY of the southbound migration curve and mean migration period range range (± std) for all species-region pairs inlcluded in our analysis.

Figure S4 Migration landmarks by species and approximate lattitude

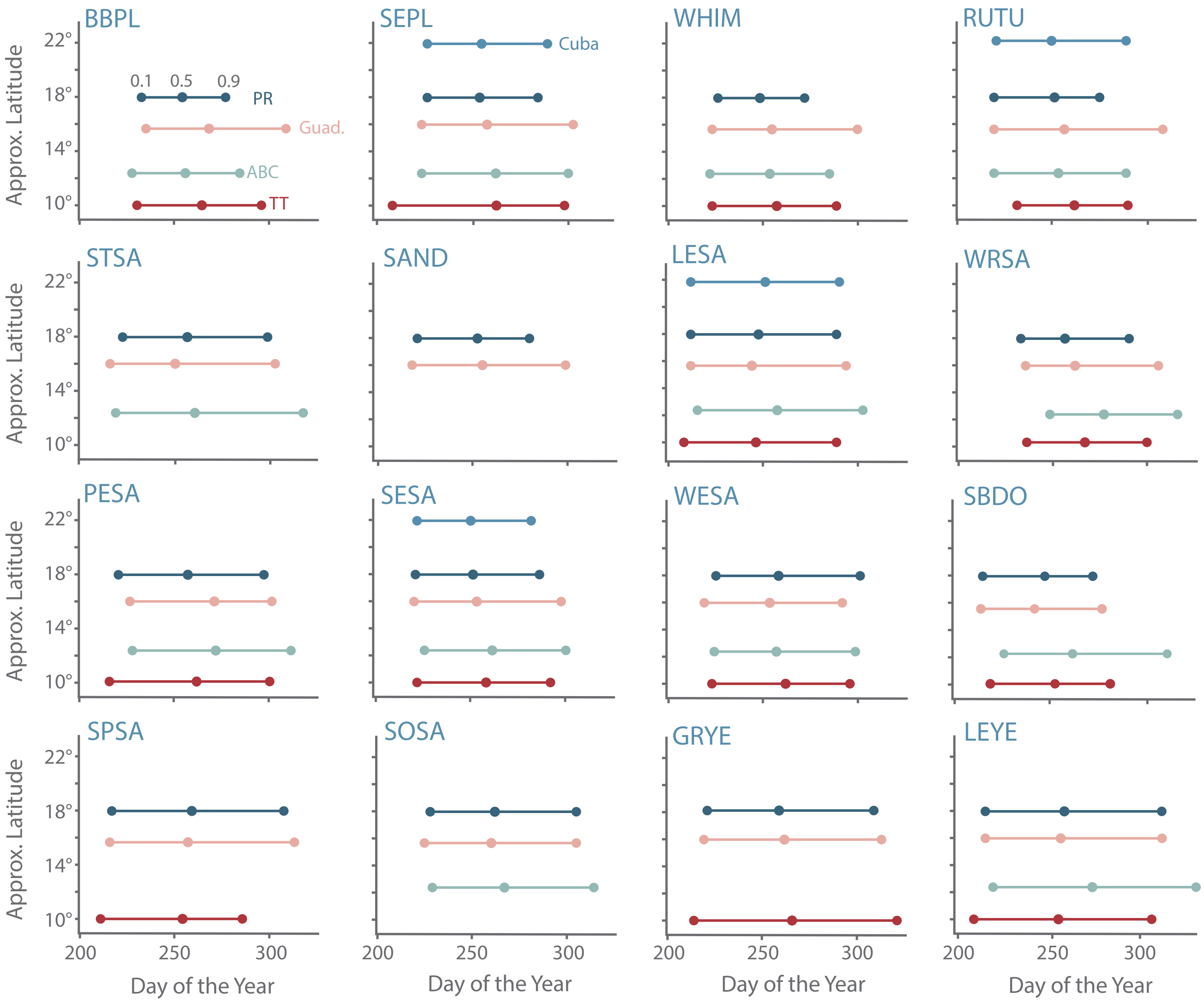

Figure S4 Migration landmarks (0.1, 0.5, and 0.9 quantiles) for each species. Regions are arranged on the y-axis by approximate lattitude.

Figure S5 Start of Migation Range (0.1 Quantile) Across Regions

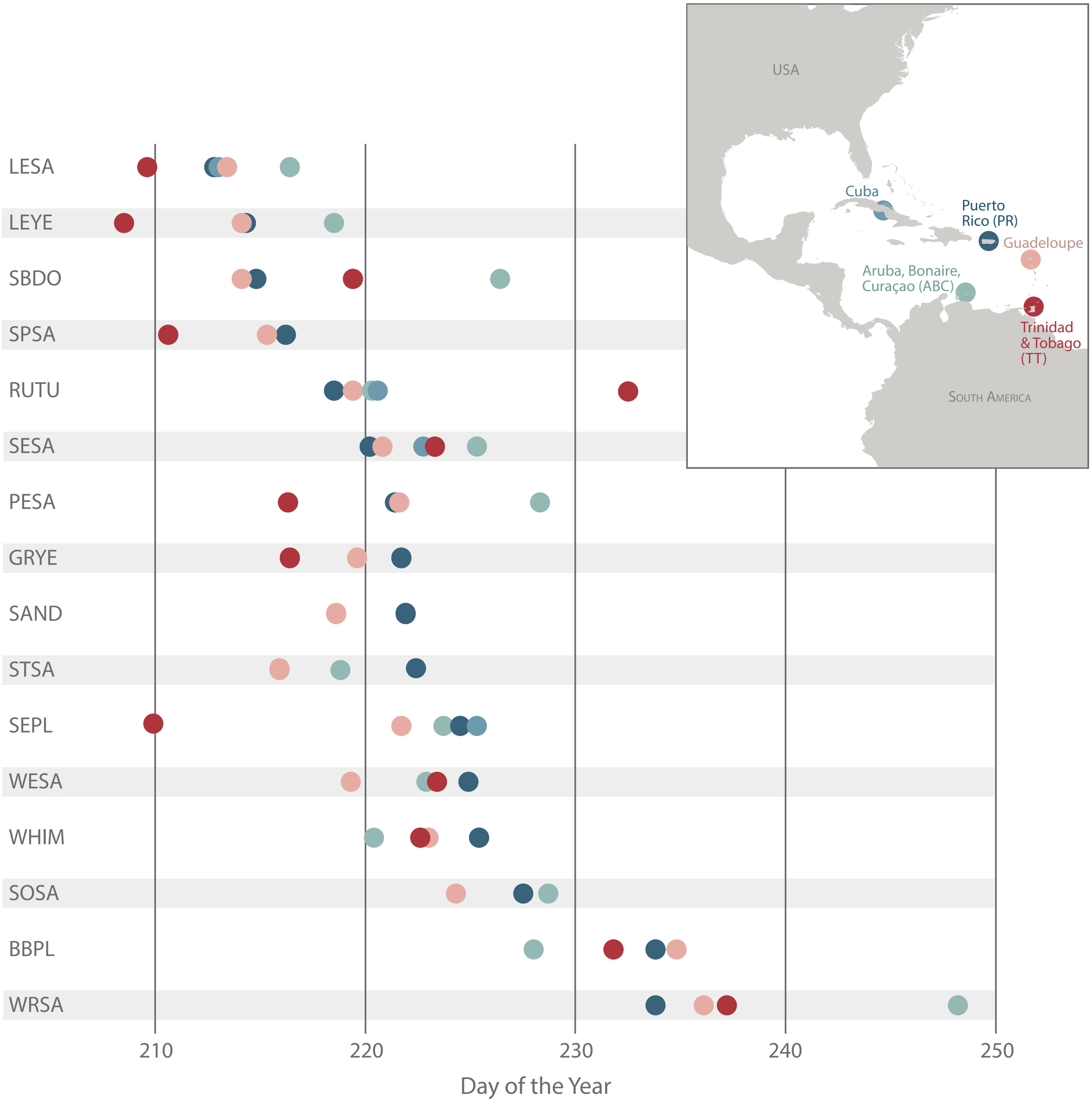

Figure S5 Day of Year (DOY) of the start of migration range (0.1 quantile) of all species-region pairs, sorted by date in PR. Despite their more southern and eastern locations, the start of migration range occurred first in TT for six species and in Guadeloupe for five species. Note not every species was analyzed for each region.

Figure S6 Overwinter and oversummer indices by region

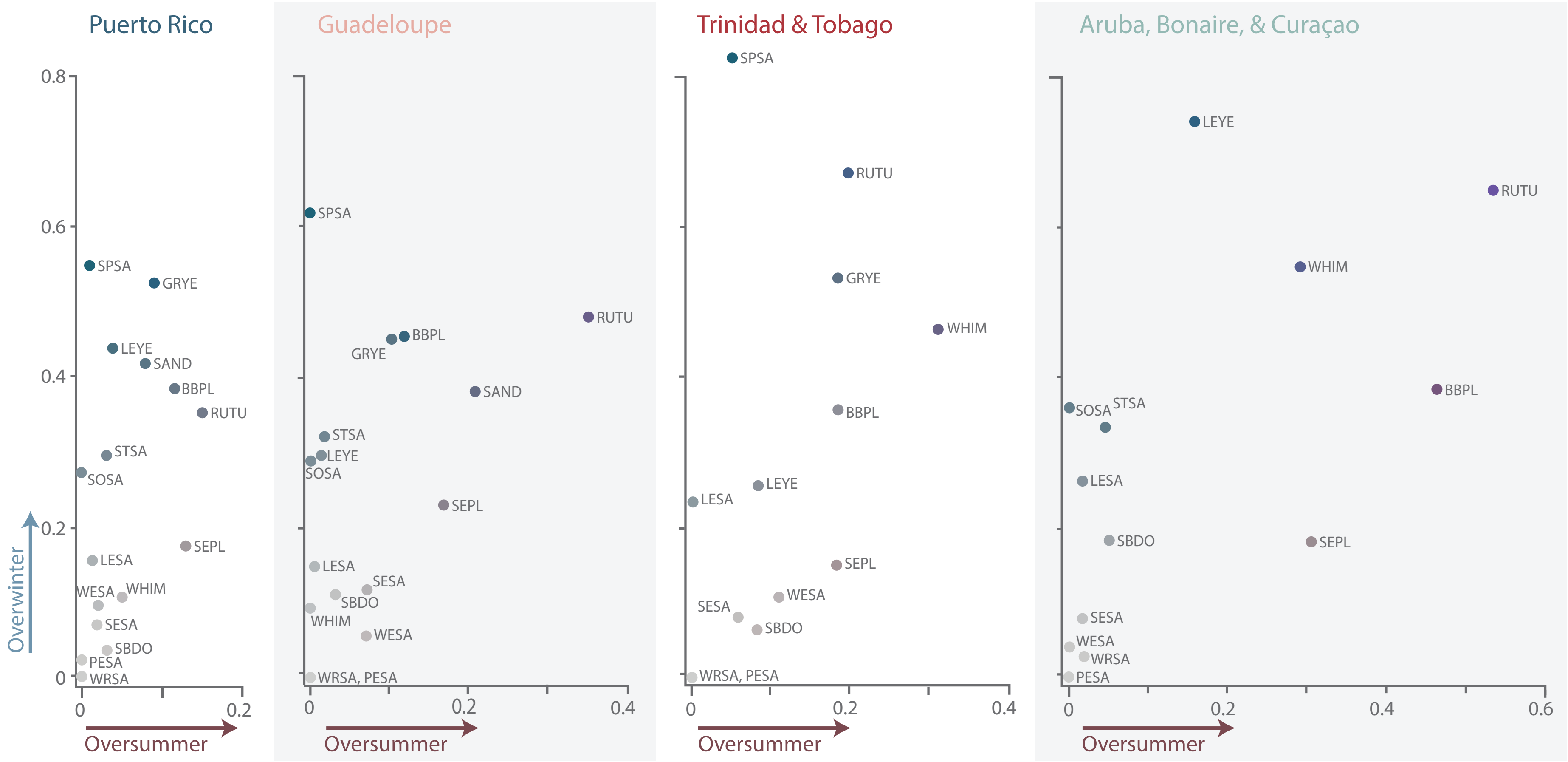

Figure S6 The winter:migration (overwinter) and summer:migration (oversummer) ratios convey how the mean proportion of presence during winter (DOY 1-25) or during summer (DOY 151-175) compares to the mean proportion of presence during the migraton midpoint (0.5 quantile DOY ± 12 days). A value close to 1 indicates that a species was recorded at similar rates during summer or winter relative to its frequency of being documented during migration; that is, some individuals appear to be using the region to overwinter or oversummer. Please note that these indices are comparisons within species-region pairs. Therefore, for example, if one species has a higher overwintering value than another in the same region, it does not necessarily imply that species is more likely to overwinter. Instead of direct numeric comparisons, we recommend interpreting these results qualitatively.

Figure S7 Examples of corresponding year-round presence data with overwinter and oversummer indices

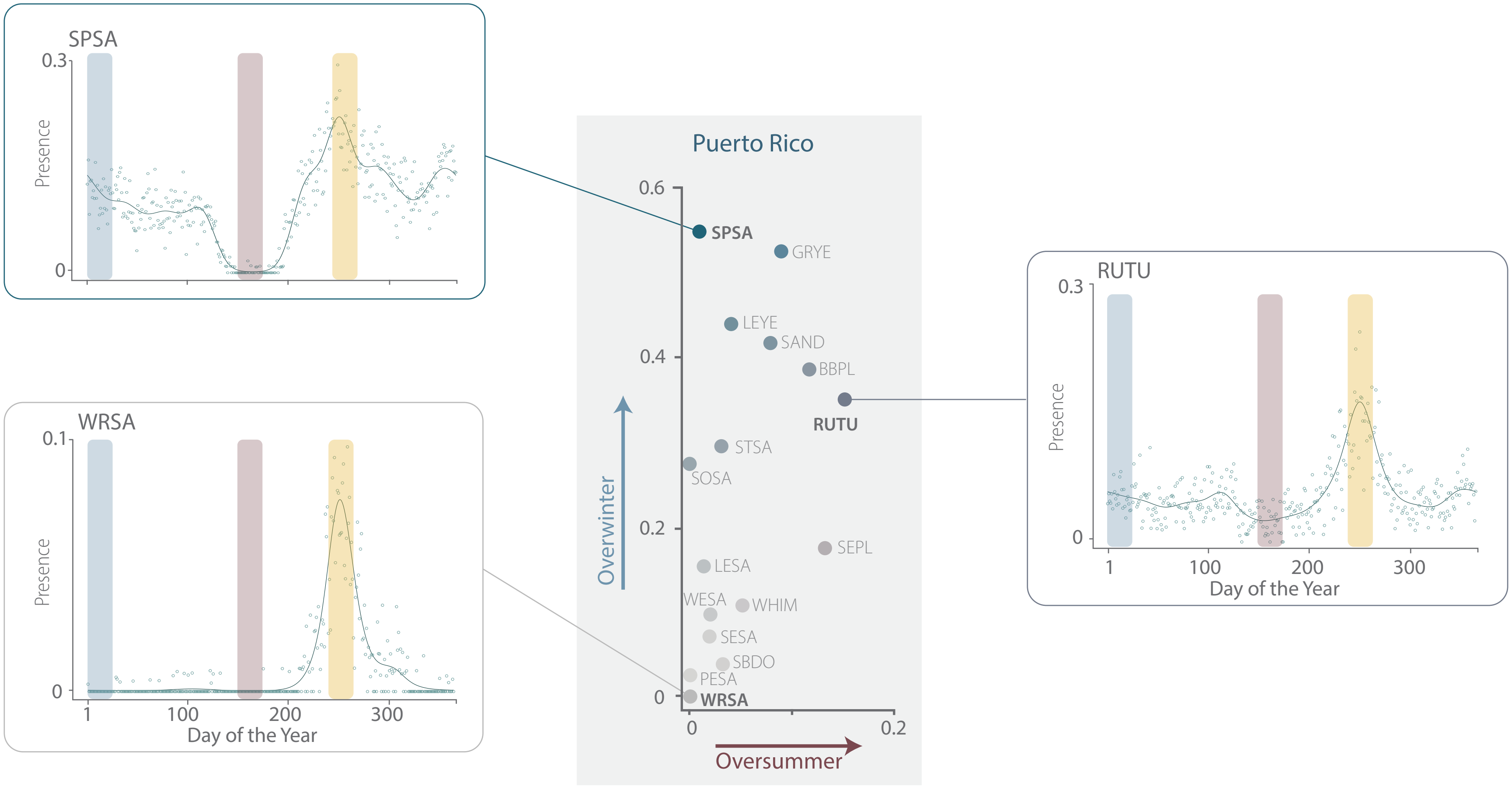

Figure S7 Cumulative (2010-2020) year-round proportion of presence data (open circles) for SPSA, WRSA, and RUTU in Puerto Rico and their overwinter and oversummer indices (center plot with gray background). Blue, red, and orange bars show approximate sampling period for winter, summer, and migration proportion of presence means. The overwinter index for SPSA was high (0.55) while the oversummer index was low (0.01). Indices were zero or close to zero for WRSA. For the RUTU, the overwinter index was 0.35 and oversummer index 0.16. See figure caption for S1.6 for description of overwinter and oversummer indices.

Figure S8 Overwinter and oversummer indices by species

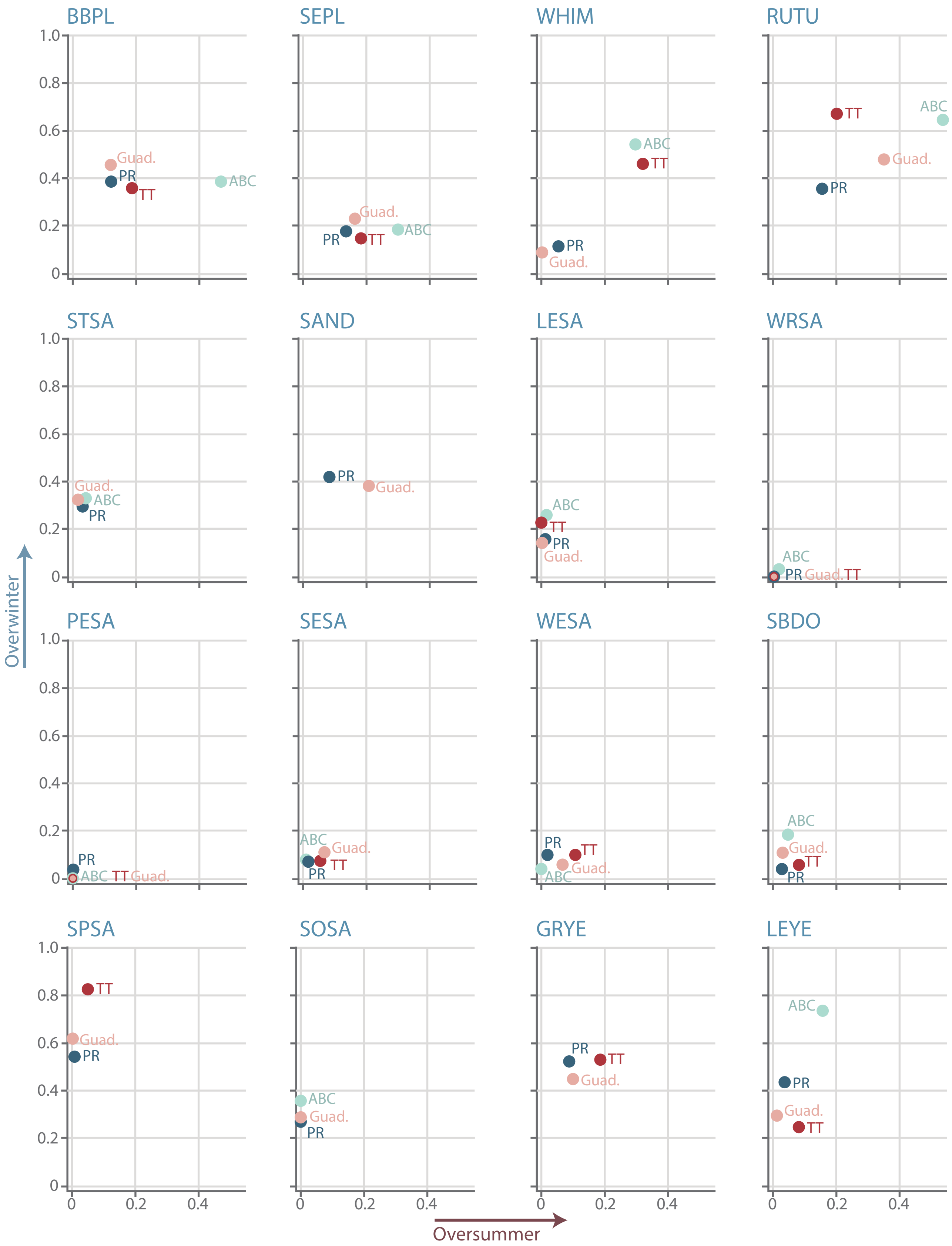

Figure S8 The winter:migration (overwinter) and summer:migration (oversummer) ratios convey how the mean proportion of presence during winter (DOY 1-25) or during summer (DOY 151-175) compares to the mean proportion of presence during the migration midpoint (0.5 quantile DOY ± 12 days). A value close to 1 indicates that a species was recorded at similar rates during summer or winter relative to its frequency of being documented during migration; that is, some individuals appear to be using the region to overwinter or oversummer. Please note that these indices are comparisons within species-region pairs. Instead of direct numeric comparisons, we recommend interpreting these results qualitatively.

Figure S9 Examples of presence and absence of end-of-year secondary model peaks

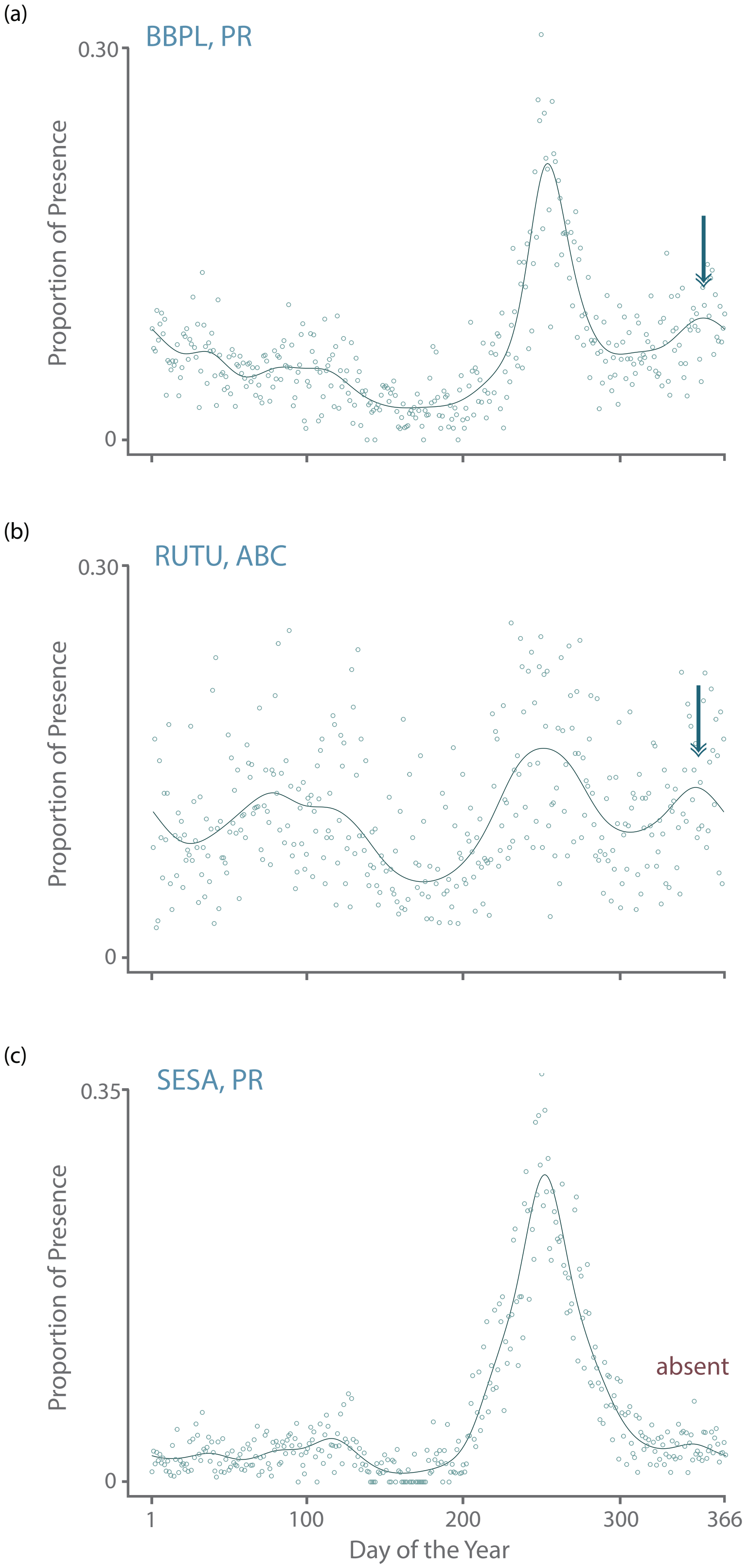

Figure S9 Cumulative (2010-2020) year-round proportion of presence data (open circles) and fitted GAM (solid line) for BBPL in PR (a) and RUTU in ABC (b) and SESA in PR (c). Smaller, secondary model peaks as indicated by the arrows in (a) and (b) were apparent for some species-regions pairs. Other species-region pairs - for example, (c) - did not show this pattern.

Figure S10 Southbound migration and hunting season in Guadeloupe

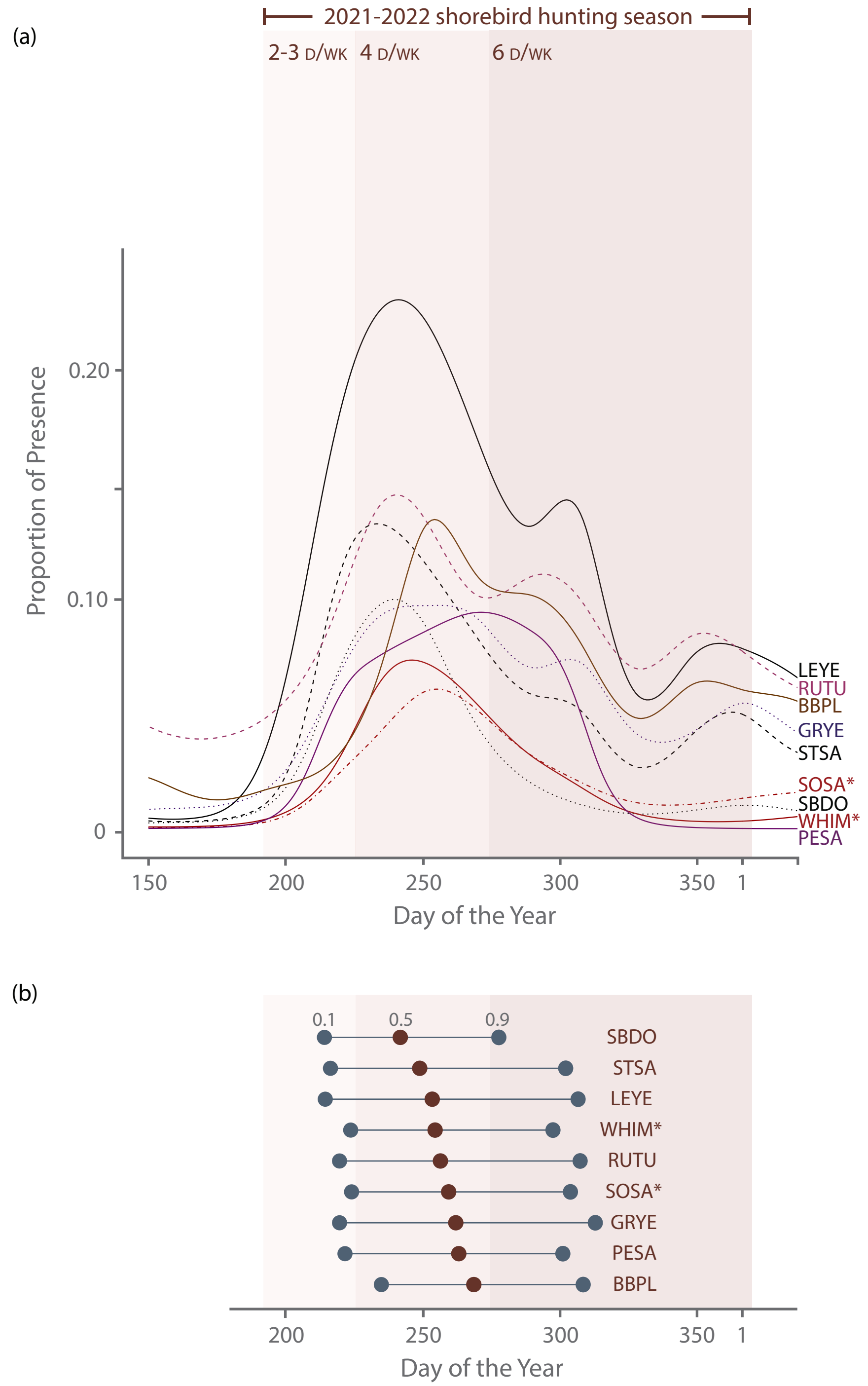

Figure S10 Current (2021-2022) Guadeloupe shorebird hunting season dates (pinks) overlaid with (a) migration curves and (b) migration landmarks of harvestable -- or previously harvestable (*) -- species included in our analysis. Days/week of legal hunting varies by date, with periods of fewer days/week enacted to conserve shorebirds. 14 Jul - 15 Aug = 2-3 days/week; 16 Aug - 30 Sep = 4 d/wk; 1 Oct - 1st Sunday in Jan = 6 d/wk. The shorebird hunting season encompasses the entire migration range (0.1 to 0.9 quantiles) for all species. Two species had most of their migration range fall within the period of fewer hunting days/week (SBDO, WHIM) while the others, such as BBPL, had about half of its migration range during the most hunting days/week. Note: WHIM hunted as recently as 2020-2021 season but hunting is prohibited during the current season. A moratorium of hunting SOSA has been in effect since 2013.

Figure S11 Examples of potential underlying pehnomena affecting proportion of presence

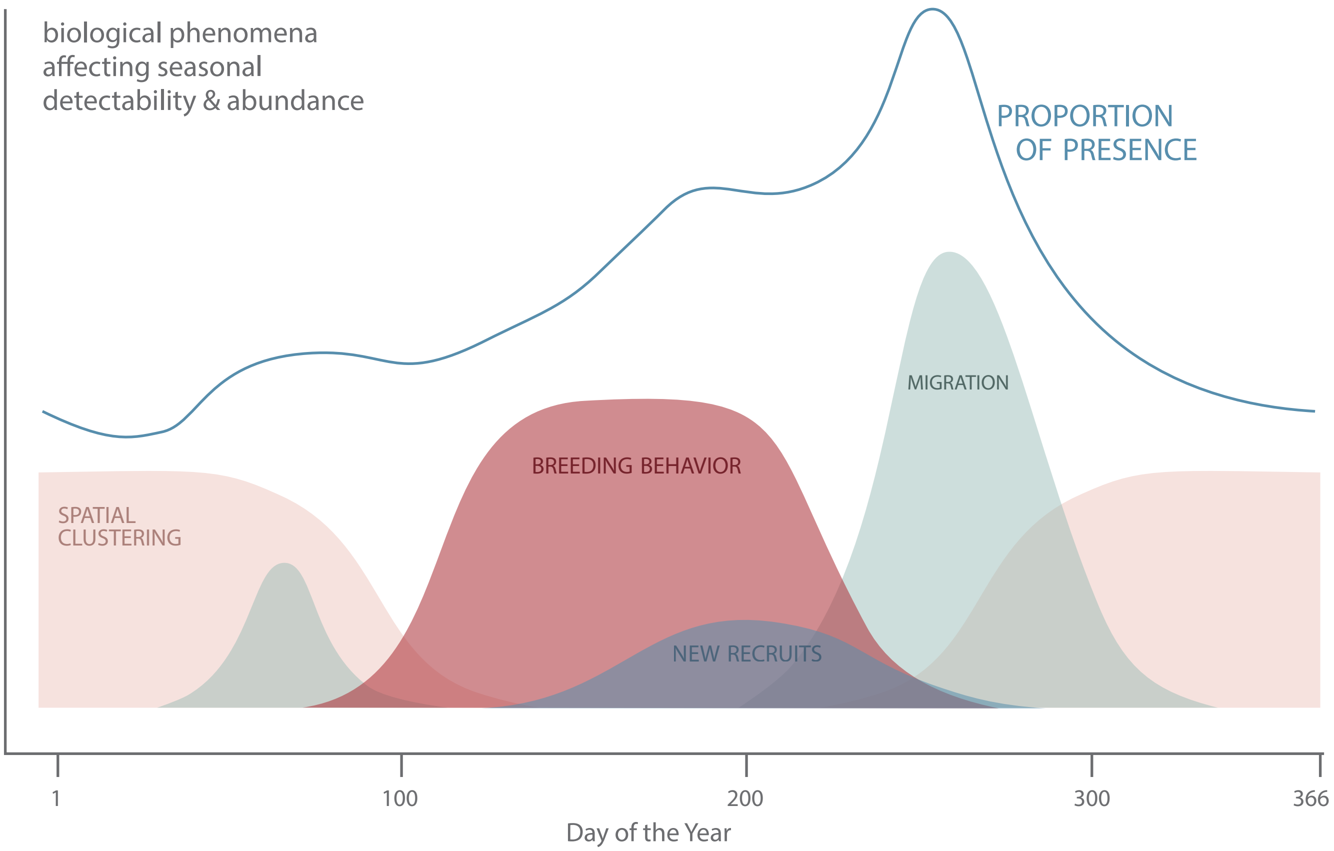

Figure S11 There are several biological phenomena, present in different intensities throughout the year, that may influence proportion of presence. Spatial clustering, breeding behavior, new recruits, and migration are some examples of such phenomena.

Figure S12 Individual and group contributions to migration curve

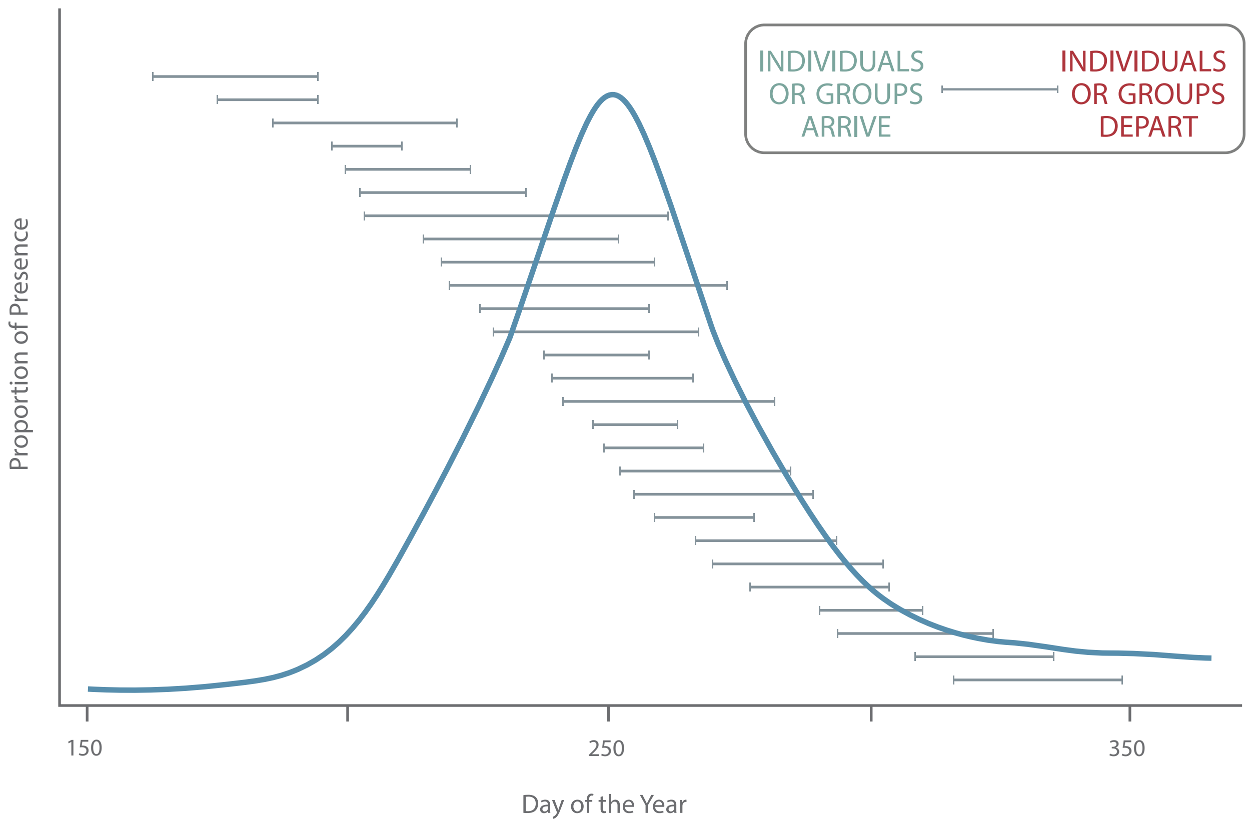

Figure S12 A hypothetical migration curve (blue solid line). Horizontal lines indicate possible arrivals and departures of individuals or groups. Birds may arrive in varying numbers, stay for varying lengths of time, and depart throughout the duration of the migration curve. The figure provides a heuristic view of the concept; migration curve was not generated from horizontal lines.

Figure S13 Total eBird Checklists per DOY (2010-2020) by region

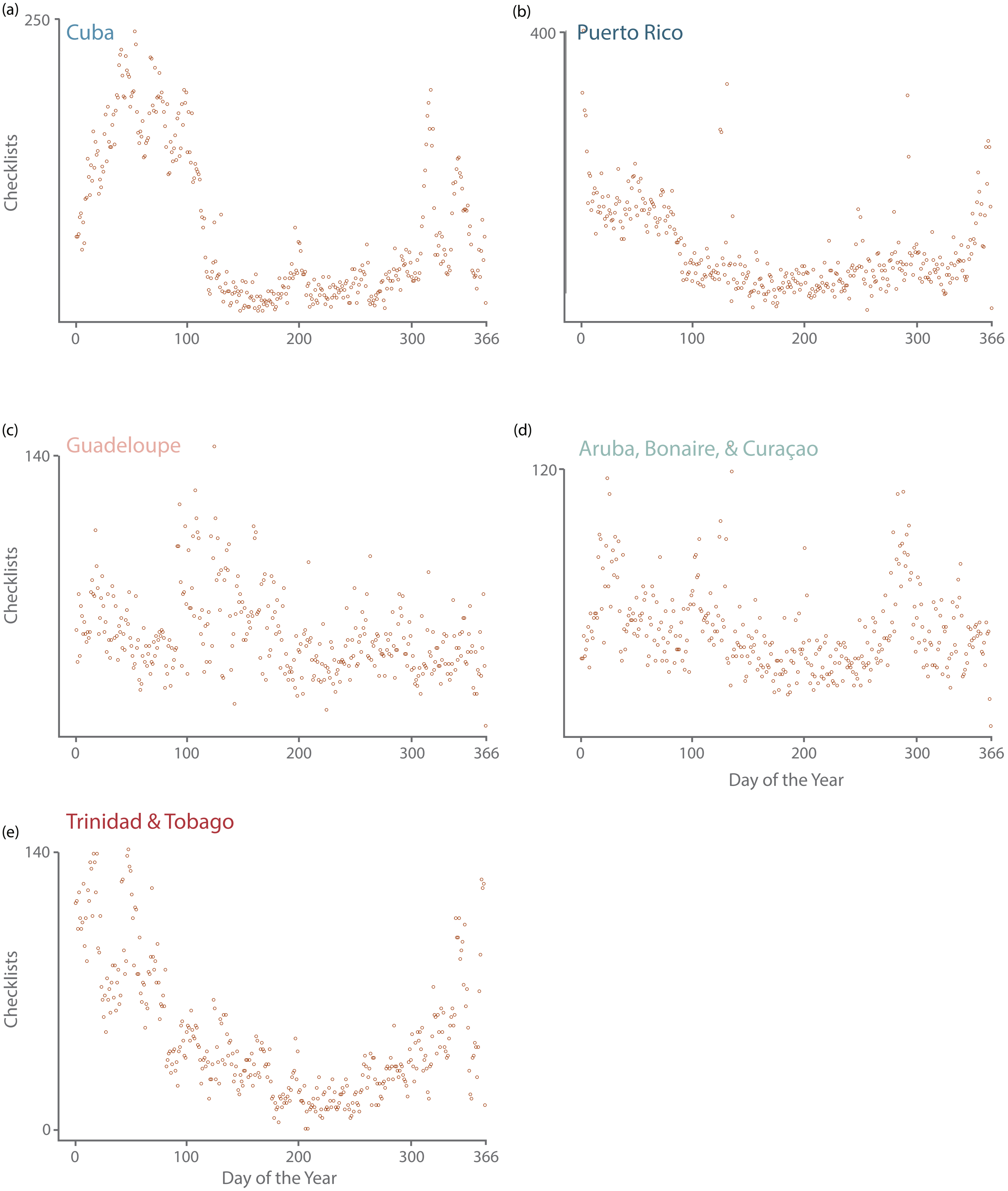

Figure S12 Cumulative number of eBird checkslists 2010-2020 per day of year (DOY) for each of the five regions.
